# Green Synthesis of Magnetic Fe_2_O_3_ Nanoparticle with *Chenopodium glaucum* L. as Recyclable Heterogeneous Catalyst for One-Pot Reactions and Heavy Metal Adsorption

**DOI:** 10.3390/molecules29194583

**Published:** 2024-09-26

**Authors:** Rahul Thakur, Navneet Kaur, Manvinder Kaur, Pradip K. Bhowmik, Haesook Han, Kishanpal Singh, Fohad Mabood Husain, Harvinder Singh Sohal

**Affiliations:** 1 Medicinal and Natural Product Laboratory, Department of Chemistry, Chandigarh University, Gharuan, Mohali 140413, Punjab, India; 2 Department of Chemistry and Biochemistry, University of Nevada Las Vegas, 4505 S. Maryland Parkway, Box 454003, Las Vegas, NV 89154, USA; 3 Department of Chemistry, Punjabi University, Patiala 147002, Punjab, India; 4 Department of Food Science and Nutrition, College of Food and Agriculture Sciences, King Saud University, Riyadh 11451, Saudi Arabia

**Keywords:** iron oxide nanoparticles, green synthesis, heterogenous catalyst, xanthene derivatives, heavy metal adsorption

## Abstract

The growth of the environment depends upon developing greener and ecological methods for managing pollutants and contamination from industrial wastewater, which causes significant effects on human health. The removal of these pollutants from wastewater using nanomaterials covers an ecological method that is free from expensive and secondary pollution. In this report, we developed magnetic iron nanoparticles from *Chenopodium glaucum* (CG), which showed excellent adsorption capacity at pH 5 for selective Hg^2+^ and Pb^2+^ metal ions among various heavy metal ions, with maximum adsorption capacities of 96.9 and 94.1%, respectively. These metals’ adsorption process conforms to the Langmuir model, which suggests that monolayer adsorption transpires on CG–Fe_2_O_3_ nanoparticles. CG–Fe_2_O_3_ nanoparticles also act as an efficient and recyclable heterogeneous catalyst for one-pot synthesis of xanthene derivatives, yielding products with high yields (up to 97%) and excellent purity (crystalline form) within a short timeframe (6 min) using microwave irradiations (at 120 W).

## 1. Introduction

Xanthenes are significant heterocycles with numerous applications in pharmaceutical chemistry [1]. Xanthene derivatives exhibit notable pharmaceutical properties, including antibacterial [2], analgesic [3], antiviral [4], anti-inflammatory [5], antimalarial [3], and anticancer activities [4]. Due to their versatility, there has been considerable interest in researching the synthesis of xanthene derivatives, which employ nanoparticles as catalysts [6]. Despite numerous studies for the catalytic synthesis of xanthene derivatives, the issue of retrieving nanocatalysts from reaction mixtures remains a challenge [2]. Recently, magnetic nanoparticles have emerged as a valuable category of nanocatalysts. Their separation process is straightforward and cost-effective, minimizing catalyst loss and improving reusability [7]. Moreover, magnetic nanoparticles exhibit high catalytic efficiency due to their extensive surface area and are relatively inexpensive and non-toxic to produce. Furthermore, green synthesis plays a crucial role in environmental protection by reducing and eliminating toxic substances and hazardous wastes. This method not only minimizes the environmental impact but also promotes sustainability by using eco-friendly materials and processes [8].

Heavy metal ions (HMIs) are the major micropollutants and have a serious impact on the environment [9,10]. The release of HMIs from agricultural or industrial activities into the environment is causing contamination and degradation and posing risks to human health [11,12]. Once released into the environment in small to trace amounts, these poisonous HMIs can persist for decades and cause serious human illnesses due to their non-biodegradable nature [13,14]. The removal of HMIs from wastewater and industrial effluents through chemical methods is effective but often not economically viable [15,16]. Traditional techniques such as atomic absorption spectroscopy, inductively coupled plasma mass spectrometry, X-ray fluorescence spectrometry, electrochemical methods, etc., for detecting HMIs often suffer from limitations such as complex sample preparation procedures and lengthy analysis times [17,18]. Many of these traditional techniques necessitate costly, cutting-edge equipment that is bulky and requires trained personnel to operate, thereby hindering in situ measurements [19,20].

Iron oxide-based nanomaterials are especially attractive for HMI removal due to their small size, high surface area, and magnetic properties [21]. As per the literature, numerous iron-based nanocomposites have been reported, such as (graphene oxide) GO-CuFe_2_O_4_ [22], Fe_3_O_4_@SiO_2_@Mel–Rh–Cu [23], NiFe_2_O_4_@SiO_2_@aminoglucose [24], MSrFeGO [25], NiFe_2_O_4_ [26], γ–Fe_2_O_3_ [27], and Fe_3_O_4_@SiO_2_–XO [28]. The synthesis of these compounds often involves the use of toxic chemicals and solvents. However, nanoparticles derived from the greener protocols are significantly important and create less waste for the environment [29]. Elham Rostamizadeh et al. developed a greener approach for synthesizing Fe_2_O_3_ nanoparticles using fruit extracts from *Cornelian cherry* [30]. K. Shankramma et al. showcased the uptake of magnetic Fe_2_O_3_ nanoparticles by *Solanum lycopersicum* (tomato) plants [31]. Their method offers simplicity, cost-effectiveness, and environmental benefits, making it a promising avenue for sustainable nanoparticle production [32].

In the present report, we have utilized recyclable green synthesized iron-oxide nanoparticles synthesized using *Chenopodium glaucum* (*C. glaucum*) extract, which provides significant surface area, efficient reactions, easy preparation, and convenient isolation through the magnet and offers advantages over traditional metal catalysts. In this study, CG–Fe_2_O_3_ nanoparticles were evaluated as a heterogeneous catalyst for synthesizing 2-amino-4-aryl-4H-benzo[g]chromene-3-carbonitrile derivatives (**4a–l**) through a one-pot reaction. Additionally, CG-Fe_2_O_3_ nanoparticles were found to be an efficient nano-adsorbent for extracting HMIs present in wastewater, thereby aiding in environmental remediation efforts.

## 2. Results and Discussion

Iron (II) oxide nanoparticles were synthesized using *C. glaucum* leaf extract. The color of the solution changed from orangish brown to black, indicating the formation of iron nanoparticles. These nanoparticles were washed three times with water and acetone and subsequently dried at 90 °C in a hot air oven to obtain purified black nanoparticles.

A mixture of ferric chloride (0.4 M) and ferrous sulfate (0.2 M) solution was reduced to Fe_2_O_3_, precipitating in the leaf extract. Phytochemicals such as flavonoids, quinones, and tannins acted as stabilizing agents during nanoparticle production in ethanol, a polar solvent. Phenols and terpenoids significantly contributed to the formation, capping, and stabilization of iron (II) oxide nanoparticles. Furthermore, these CG–Fe_2_O_3_ nanoparticles were characterized by FT-IR, SEM, EDS-SEM, XRD, and VSM analysis.

### 2.1. Characterization of CG–Fe_2_O_3_ Nanoparticles

#### 2.1.1. FT-IR Characterization

**Figure 1:**
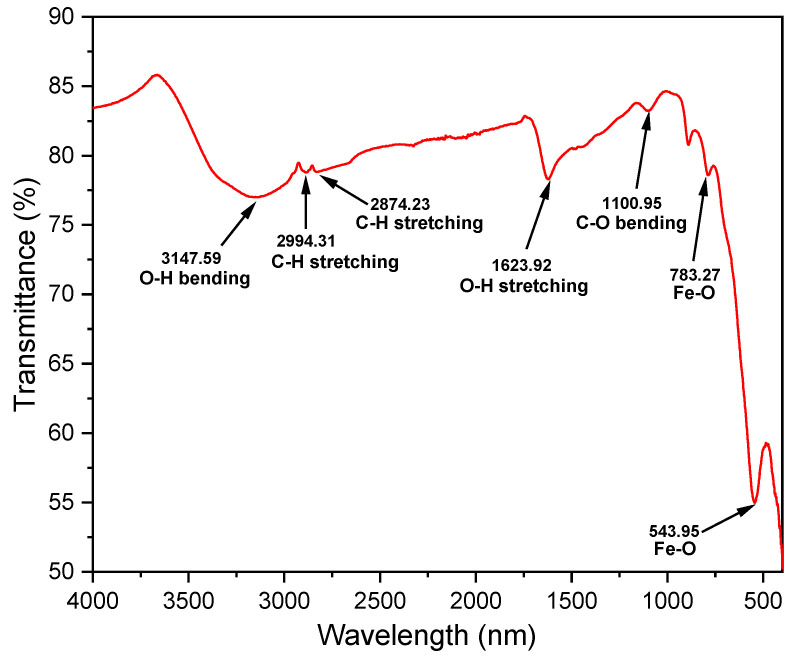
FT-IR spectra of CG–Fe_2_O_3_ NPs.

FT-IR analysis of CG–Fe_2_O_3_ NPs, presented in **Figure 1**, revealed characteristic peaks at 3147.59 cm^−1^ (absorbed water bending vibration) and 1623.92 cm^−1^ (surface hydroxyl and O-H stretching modes), confirming the presence of iron oxide nanoparticles. Prominent peaks at 783.27 cm^−1^ and 543.95 cm^−1^ further validate the Fe–O bond, consistent with previous studies [33,34], ensuring successful synthesis without significant impurities. Moreover, the presence of phytochemicals was confirmed by the presence of peaks at 2994.31 cm^−1^, 2874.23 cm^−1^, and 1100.95 cm^−1^, which attributed to two C–H and one C–O bonds present in *C. glaucum*.

#### 2.1.2. SEM Characterization

**Figure 2:**
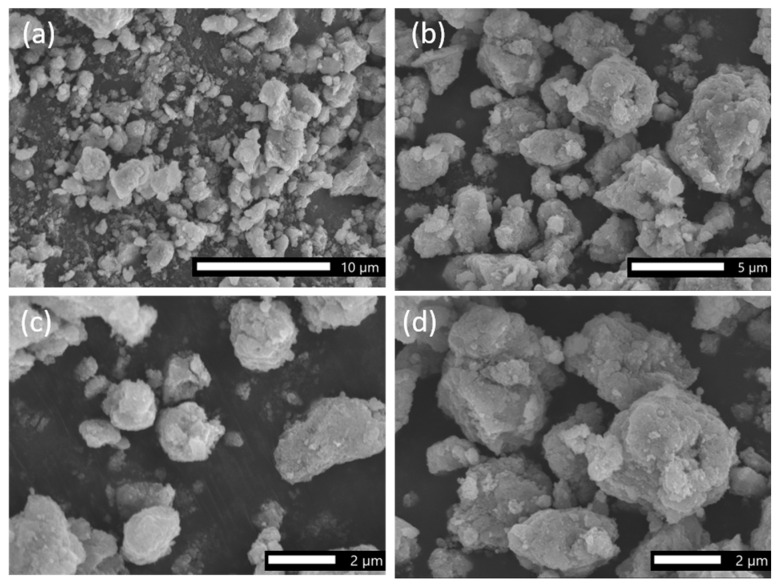
(**a**–**d**). SEM images of magnetic CG–Fe_2_O_3_ NPs.

The FE-SEM images of CG–Fe_2_O_3_ NPs are depicted in **Figure 2a–d** at varying magnifications from 500 to 7500 times, revealing a morphology consistent with nanoparticles. The SEM images indicate that the CG–Fe_2_O_3_ NPs exhibit spherical forms with rough surface textures. However, there is a lack of uniformity in both size and shape, resulting in a broad size distribution.

**Figure 3:**
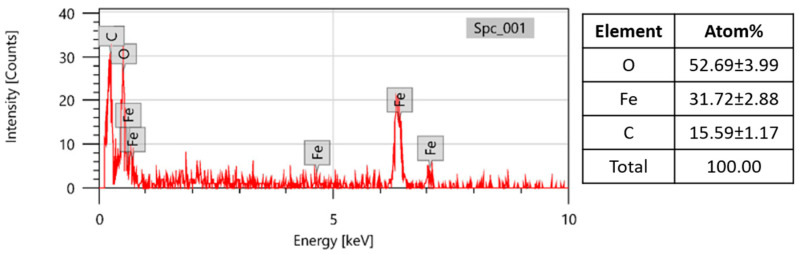
EDS-SEM image of magnetic CG–Fe_2_O_3_ NPs.

Energy dispersive X-ray spectroscopy (EDS) analysis, presented in **Figure 3**, confirmed the elemental composition of synthesized CG–Fe_2_O_3_ NPs, displaying prominent peaks corresponding to iron and oxygen. Analysis revealed a composition of 31.72 ± 2.88 atom% iron, 52.69 ± 3.99 atom% oxygen, and 15.59 ± 1.17 atom%, consistent with CG–Fe_2_O_3_ NPs. The data closely matched theoretical calculations, indicating uniform composition [35]. Sharp peaks between 0–1 KeV and 6–8 KeV confirmed the crystalline nature, accounting for 55.1% of CG–Fe_2_O_3_ NPs (**Figure S2**, in the Supplementary Materials).

#### 2.1.3. XRD Characterization

**Figure 4:**
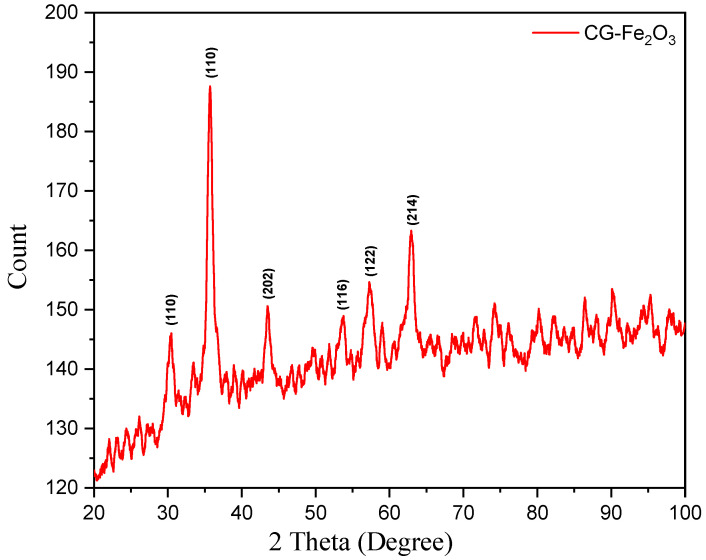
XRD pattern of CG–Fe_2_O_3_ NPs.

Confirmation of the successful synthesis of CG–Fe_2_O_3_ NPs was achieved through powder X-ray diffraction (XRD). The obtained diffraction pattern lacked sharp peaks, indicating that the synthesized nanoparticles were not well crystallized (**Figure 4**). However, a few weak peaks were observed at 2θ values of 30.46, 35.72, 43.47, 53.77, 57.25, and 62.94, corresponding to the (110), (110), (202), (110), (122), and (214) planes of CG–Fe_2_O_3_, respectively. The crystallite size of 9.05–11.40 nm was estimated by using the Scherrer formula.

#### 2.1.4. TEM Characterization

**Figure 5:**
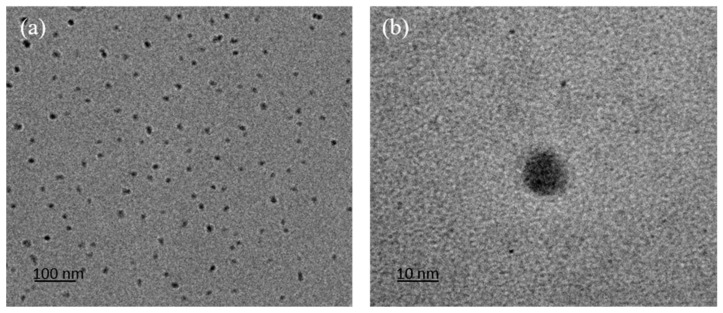
TEM image of magnetic CG–Fe_2_O_3_ NPs (**a**) low-magnification, and (**b**) high-magnification.

The transmission electron microscopy (TEM) images of CG–Fe_2_O_3_ nanoparticles in **Figure 5** revealed particle sizes ranging from 10 to 100 nm. Specifically, **Figure 5a** displayed the low-magnification view which shows small, dot-like nanoparticles that are well-dispersed across the field, suggesting minimal aggregation and possibly surface modifications or stabilizing agents preventing clumping. The particles are uniform, indicating controlled synthesis conditions. Whereas **Figure 5b** presented high-magnification images, showing a single nanoparticle with smooth edges, likely indicating a well-defined structure. The darker central region could suggest a denser core or core-shell morphology, though surface features appear smooth with no significant irregularities. The interpretation of these TEM images indicated crystallite sizes in the range of 9–11 nm, consistent with the results obtained from XRD analysis [36].

#### 2.1.5. VSM Characterization

**Figure 6:**
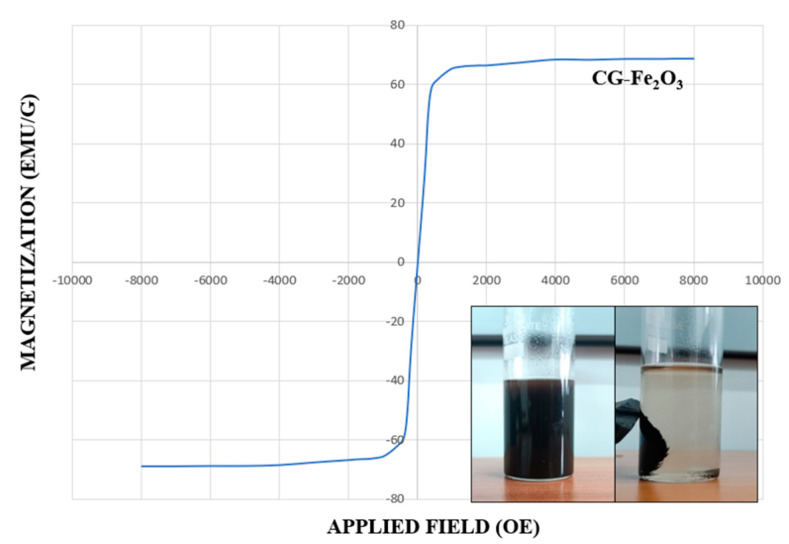
VSM characterization of CG–Fe_2_O_3_ NPs.

The magnetic property of Fe_2_O_3_ was assessed using VSM at 25 °C (**Figure 6**). CG–Fe_2_O_3_ NPs exhibited a saturation magnetization (M_s_) of 68.8 emu/g. CG–Fe_2_O_3_ NPs exhibit good magnetic response and can be easily attracted with the help of an external magnetic field. Notably, their low coercivity (H_c_) and magnetic remanence (M_r_) values—specifically 25.5 and 1.873, respectively—indicated superparamagnetic-like behavior. Furthermore, the M_r_/M_s_ ratio for CG–Fe_2_O_3_ NPs was 0.027, suggesting their proximity to acting as superparamagnetic materials [37].

### 2.2. Influence of Substituents on the Synthesis of 2-Amino-4-aryl-4H-benzo[g]chromene-3-carbonitrile Derivatives (***4a**–**l***)

The developed optimized method offers efficiency by yielding a significant amount of desired product quickly while also being environmentally friendly. Therefore, we progressed to the subsequent phase of our research, exploring the adaptability of the process with various substrates of aldehyde. We conducted the condensation by a series of aromatic and hetero-aromatic aldehydes to gauge its versatility using the same protocol (**Table 1**). These reactions continue effortlessly, even in the presence of numerous EDG (–Me and –OMe) as well as EWG (–NO_2_) on aldehyde, enabling effective reactions. The efficacy of this approach is remarkable, yielding high percentages (93.6–99.1%) for the final products.

Since the reactivity of the aldehyde is influenced by the groups attached to it, we concluded from the data in **Table 1** that electron-withdrawing groups improve the electrophilicity of the carbonyl carbon, which makes it further active to undergo a nucleophilic attack by the malononitrile anion from malononitrile. The impact of negative inductive effects becomes evident with the comparison between the yields and reaction periods of products gained from 4-nitrobenzaldehyde (**4c**), 2-chlorobenzaldehyde (**4d**), and 4-chlorobenzaldehyde (**4e**) as substrates. Conversely, 2-hydroxybenzaldehyde (**4h**), and 2-thiophenyl benzaldehyde (**4i**) displayed lower reactivity, as indicated by prolonged reaction times. This discrepancy is attributed to the positive mesomeric effect induced by the OH group in 2-hydroxybenzaldehyde (**4h**).

### 2.3. Characterization of 2-Amino-4-phenyl-4H-benzo[g]chromene-3-carbonitrile Derivative (***4a***)

The confirmation of the formation of the desired product was done with spectroscopic techniques such as FT-IR, ^1^H, and ^13^C NMR. In the case of compound **4a**, the FT-IR spectra exhibit notable absorptions at 2182.36 cm^−1^, indicating stretching vibrations of the C≡N, whereas 3338.00 cm^−1^ indicates N–H stretching vibrations of –NH_2_. Peaks corresponding to the stretching of sp^2^ hybridized C–H bonds were detected at 3432.74 cm^−1^, and that of C=C aromatic stretching vibrations were seen in the range of 1589.45 cm^−1^. In the ^1^H NMR spectra (500 MHz, CDCl_3_) of compound **4a**, two singlets were detected at δ 6.98 and 5.30 ppm, corresponding to –NH_2_ and C4–H, respectively. The peaks in the range of 7.14–7.95 ppm appeared due to aromatic protons. In ^13^C NMR (500 MHz, CDCl_3_), the most downfield peak was observed at δ 159.61 ppm, corresponding to the C-3 carbon. Other peaks observed include δ 146.74 (C-2) and 57.93 (C-4), indicating sp^3^ carbons in the structure. Additionally, peaks were observed at δ 115.58 ppm, corresponding to the C≡N group, and in the aromatic region at δ 124.83–145.60 ppm (ArC-5 to 14) and δ 116.69–123.52 ppm (ArC-1′ to 6′), representing the aromatic carbons. This observation confirms the formation of compound **4a**.

### 2.4. Environmental Performance Metrics

The environmental implications of synthetic routes were evaluated in this study using two mass-based systems of measurement: atom economy and E-factor [6]. Atom economy, representing the efficiency of a chemical reaction, was calculated by dividing the molecular weight of the required products **4a**–**l** by the total molecular weight of all reactants, i.e., *β*-naphthol **1**, substituted benzaldehyde **2a**–**l**, and malononitrile **3**. The resulting high atom economy values, typically over 94%, indicated substantial integration of starting materials into the final product with negligible waste generation, despite the elimination of a water molecule [38]. The atom economy of compound **4a** is illustrated in **Equation (1)** as given below:

(1) $$\begin{array}{ll} Atom\;Economy & \left(\%\right)=\frac{Molecular\;weight\;of\;desired\;product}{Molecular\;weight\;of\;all\;the\;reactants\;used}\times 100\\ & =\frac{298\;g/mol}{(144.16+66.06+106.12)\;g/mol}\times 100=94.20\% \end{array}$$

Additionally, the E-factor, which considers waste production per kilogram of product, was computed to assess environmental suitability. Most synthesized products **4a**–**l** exhibited E-factor values close to zero, indicating minimal waste generation attributed to catalyst recyclability and greener solvent conditions. These findings underscored the overall sustainability of the synthetic processes examined. The E-factor of compound **4a** is depicted in **Equation (2)** as given below:

(2) $$E\;factor=\frac{Mass\;of\;the\;waste}{Mass\;of\;the\;desired\;product}=\frac{0.063\;g}{1.427g}=0.04$$

### 2.5. Adsorption of Heavy Metal Ions

#### 2.5.1. Effect of Adsorbent Dose on the Removal of Heavy Metal Ions

**Figure 7:**
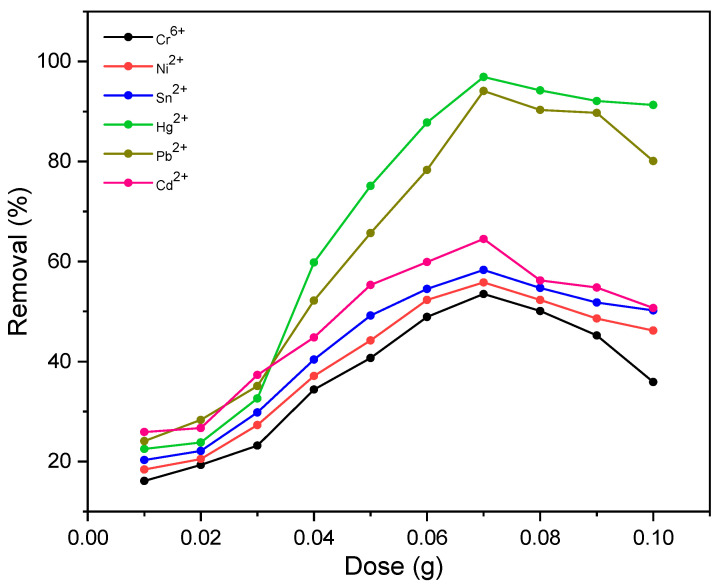
Effect of adsorbent dose (CG–Fe_2_O_3_ NPs) on Cr^6+^, Ni^2+^, Hg^2+^, Sn^2+^, Cd^2+^, and Pb^2+^ metals ions removal.

The experiments on adsorbent dose variation were conducted to determine the optimal dose for CG–Fe_2_O_3_ NPs when treating the Cr^6+^, Ni^2+^, Hg^2+^, Sn^2+^, Cd^2+^, and Pb^2+^ metals solution at a concentration of 100 mg/L for a contact time of 60 min at temperature 303 K. The adsorbent quantities ranged from 0.01 g to 0.10 g in a 100 mL solution volume. As depicted in **Figure 7**, the removal percentage of metal ions increased with the increase in dose until reaching 0.07 g, achieving a maximum removal of 96.9 and 94.1% for Hg^2+^ and Pb^2+^, respectively, using CG–Fe_2_O_3_ NPs. Beyond this dosage, there was negligible improvement observed. On the other hand, it was noticed that except for Hg^2+^ and Pb^2+^ metal ions, all other metal ions were removed insignificantly in the adsorption studies. Consequently, 0.07 g was identified as the optimal amount for future studies on Hg^2+^ and Pb^2+^ metal ions removal using CG–Fe_2_O_3_ NPs.

#### 2.5.2. Effect of pH on Removal of Heavy Metal Ions

**Figure 8:**
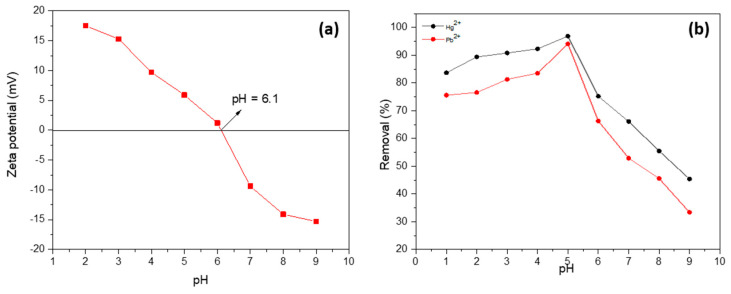
(**a**) Zero-point charge of CG–Fe_2_O_3_ NPs. (**b**) Effect of pH on removal of Hg^2+^ metal ion by CG–Fe_2_O_3_ NPs.

The adsorption experimentations for Cr^6+^, Ni^2+^, Hg^2+^, Sn^2+^, Cd^2+^, and Pb^2+^ metal ions removal using CG–Fe_2_O_3_ NPs were conducted by varying the pH from 2 to 10 while keeping all other parameters constant. As depicted in **Figure 8b**, the impact of pH on adsorption behavior was examined. It was observed that the adsorption capacities of Hg^2+^ and Pb^2+^ metal ions on CG–Fe_2_O_3_ NPs increased with rising pH levels. By using HCl and NaOH, the pH of the solution was controlled. It was noted that the metal solution exhibited maximum adsorption at pH 5. The CG–Fe_2_O_3_ NPs were found to have a zero-point charge of 6.1, as shown in **Figure 8a**, as determined from zeta potential studies conducted across various pH levels. At the pH equal to the zero-point charge (pHzpc), the surface charge of the adsorbent is neutral. Above this pHzpc, the surface becomes negatively charged, while below it, the surface becomes positively charged. Consequently, above the pHzpc, cation adsorption takes place through electrostatic attraction, whereas below the pHzpc, anion adsorption occurs due to the negatively charged surface of the adsorbent. It was noted that the removal efficiency increases as the pH decreases from the pHzpc to pH 5. However, beyond pH 5, the removal efficiency starts to decrease again. In strongly acidic conditions, the adsorption capacity diminishes once more.

#### 2.5.3. Isotherm Studies

Adsorption experiment batches were conducted with constant parameters such as a CG–Fe_2_O_3_ NPs dose of 0.07 g metal ions in a 100 mL solution, pH of 5, and temperature of 303 K to assess the adsorption capacity at varying metals concentrations, from 10 to 100 mg/L of Hg^2+^ and Pb^2+^. As depicted in **Figure 7**, it is evident that the adsorption capacity increases with the increase in the concentration of the metal ions solution and shows maximum adsorption capacity at 70 mg/L. Beyond this concentration, further rises to 100 mg/L result in a minor improvement in the adsorption capacity.

Furthermore, the experimental data were fitted to gain insights into the mechanism of heavy metal ions adsorption, using Langmuir’s and Freundlich’s isotherm models. The mathematical expressions for the nonlinear Langmuir’s and Freundlich’s isotherm models are represented by **Equations (3) and (4)**, respectively.

(3) $$q_{e}=\frac{q^{0}bC_{e}}{1+bC_{e}}$$

(4) $$\log q_{e}=\log K_{F}+\frac{1}{n}\log C_{e}$$

where q_e_ (mg/g) and q^0^ (mg/g) represent equilibrium and maximum adsorption capacity, respectively, b (L/mg) represents Langmuir’s constant, C_e_ (mg/L) represents the equilibrium concentration, and K_F_ (mg/g) & n represents Freundlich’s constant.

**Figure 9:**
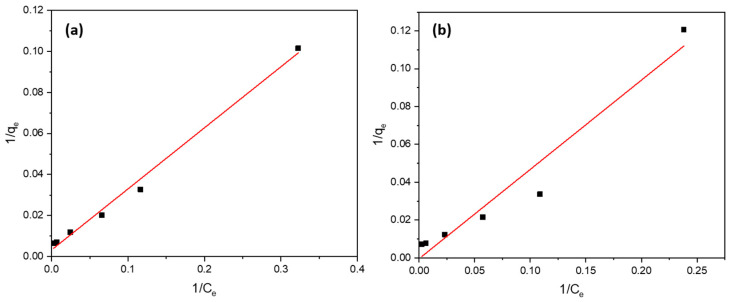
Langmuir’s isotherm plots of (**a**) Hg^2+^, and (**b**) Pb^2+^.

**Figure 10:**
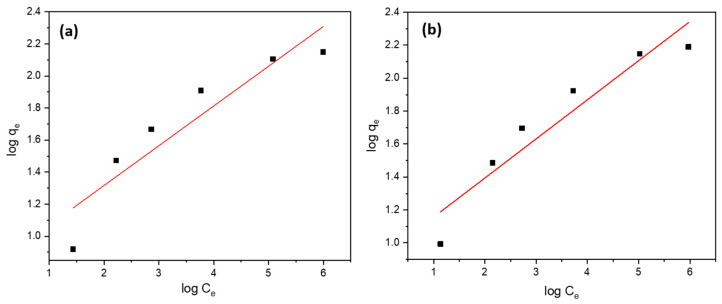
Freundlich’s isotherm plots of (**a**) Hg^2+^, and (**b**) Pb^2+^.

**Figure 9** and **Figure 10** illustrate both the nonlinear Langmuir and Freundlich curves, while **Table 2** summarizes the calculated values. The regression coefficient values imply that the adsorption process of Hg^2+^ and Pb^2+^ metals obey Langmuir’s model, indicating monolayer adsorption on the surface of CG–Fe_2_O_3_ NPs.

To assess the viability of the adsorption process, R_L_ values are calculated using **Equation (5)** derived from the Langmuir isotherm model. Values falling between 0 and 1 indicate the nature and feasibility of the adsorption process (**Table ST9**, in the Supplementary Materials).

(5) $$R_{L}=\frac{1}{1+bC_{0}}\;$$

Furthermore, based on **Table 2**, it was also concluded that the adsorption experiments for Hg^2+^ and Pb^2+^ metal ions followed the Langmuir model, with a higher R^2^ value. Moreover, CG–Fe_2_O_3_ NPs exhibited greater adsorption for Hg^2+^ and Pb^2+^ ions with 96.9% and 94.1% of maximum adsorption capacity, respectively. This obtained maximum adsorption capacity value of CG–Fe_2_O_3_ NPs was then compared with those of other reported adsorbents for Hg^2+^ and Pb^2+^ metal ions removal, as represented in **Table 3**. It is evident that CG-Fe_2_O_3_ is an effective nano-adsorbent for the adsorption of Hg^2+^ and Pb^2+^ metal ions.

## 3. Materials and Methods

### 3.1. Chemicals

All chemicals utilized in this study were procured from Sigma-Aldrich (Ludhiana, Punjab, India) and were employed without further purification. Solvents were sourced from Loba Chemie (Ludhiana, Punjab, India). The *Chenopodium glaucum* L. leaves were collected locally from Kharar, Punjab, India [30.7499° N, 76.6411° E].

### 3.2. Analytical Instruments

The melting points of synthesized products were determined using a digital melting point apparatus *via* the open capillary method. ^1^H-NMR and ^13^C-NMR spectra were recorded on a Bruker Avance NEO 500 MHz with CDCl_3_ as the solvent, at 300 and 400 MHz, respectively, referencing chemical shifts (δ) to tetramethyl silane (TMS) as an internal standard (Punjab University, Chandigarh, Punjab, India). FT-Infrared (FT-IR) spectra were obtained using the attenuated total reflection (ATR) mode on a Perkin Elmer Spectrum II instrument (Chandigarh University, Gharuan, Punjab, India). The thin-layer chromatography (TLC) technique, coupled with visualization in a UV chamber, monitored reaction progress and assessed compound purity. The magnetic characteristics of the adsorbents were assessed using a vibrating sample magnetometer, 7410 Series, Lake Shore (Punjab Agricultural University, Ludhiana, Punjab, India). The XRD analysis was conducted using a Bruker D8 Advance model (Chandigarh University, Gharuan, Punjab, India). The SEM analysis was performed using a JSM IT500 model, with magnification capabilities from × 30 to × 300,000, a display size of 128 mm × 96 mm, a tungsten filament electron gun with fully automatic alignment, and an accelerating voltage range of 0.3 kV to 30 Kv (Chandigarh University, Gharuan, Punjab, India). The zero point charge (ZPC) and the hydrodynamic dimensions of the particles were measured using a Zetasizer Nano-ZS90 instrument (Chandigarh University, Gharuan, Punjab, India). Metal ion concentrations were determined using an inductively coupled plasma optical emission spectrometer, ICP-OES, 5110 ICP-OES, Agilent Technologies (Punjab Agricultural University, Ludhiana, Punjab, India). The microwave irradiations were generated using an Anton Par monowave 200 model (Chandigarh University, Gharuan, Punjab, India).

### 3.3. Preparation of C. glaucum Extract

*C. glaucum* leaves were harvested, cleaned, and air-dried in the shade at room temperature for 6–7 days. The dried leaves were subsequently milled into a fine powder. An ethanolic extract of the plant material was prepared through maceration by mixing 10 g of the powdered leaves with 100 mL of ethanol in a 250 mL beaker, which was stirred continuously at room temperature for 24 h. Then, the subsequent extract was filtered and stored in an airtight glass container at 4 °C for later use.

### 3.4. Synthesis of CG-Fe_2_O_3_ Nanoparticles

A mixture of ferric chloride (0.4 M) and ferrous sulfate (0.2 M) solution was taken under a nitrogen atmosphere and stirred for 15 min at 70 °C. *C. glaucum* was then slowly added until the pH reached 11. The precipitates were filtered and washed with acetone and water until the pH was 7. They were dried at 70 °C for 24 h and then calcined at 500 °C for 5 h to obtain CG–Fe_2_O_3_ nanoparticles [32].

### 3.5. Synthesis of 2-Amino-4-aryl-4H-benzo[g]chromene-3-carbonitrile Derivatives (***4a**–**l***)

**Scheme 1:**
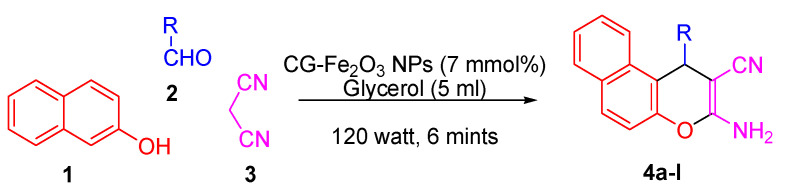
Synthesis of 2-amino-4-aryl-4H-benzo[g]chromene-3-carbonitrile derivatives (**4a**–**l**) under MW irradiations at 120 watt for 6 min.

After the optimization of reaction conditions (discussed in the Supplementary File in Section S2. Results and Discussion), a mixture composed of *β*-naphthol **1** (5 mmol), substituted benzaldehyde **2a**–**l** (5 mmol), and malononitrile **3** (5 mmol), in the presence of CG–Fe_2_O_3_ NPs (7 mmol%), was subjected to MW irradiation at 120 watt for 6 min in glycerol (5 mL) (**Scheme 1**). After completion of the reaction, magnetic nanoparticles were recovered *via* magnet, filtered off the solution, and then washed with distilled water, later, recrystallization of the resulting crude product was done by using ethanol to afford the pure crystals with an efficiency of 90–95%. All reactions were monitored using TLC with silica gel-coated plates (hexane: ethyl acetate/8:2/*v*:*v*).

### 3.6. Adsorption Experiment

Adsorption experiments were conducted using a water bath cum shaker at a speed of 300 rpm at room temperature (303 K) in 100 mL shaker flasks. The adsorbent dose and pH of solutions were investigated for the adsorption experiments. The dose of the adsorbent was kept at 0.07 g/L for all the adsorption experiments. To prevent the formation of insoluble metal hydroxides, the pH was kept at 5 for Hg^2+^ and Pb^2+^, as recommended by the above observations. pH adjustments during the experiments were achieved using either 0.1 mol/L HCl or 0.1 mol/L NaOH solutions. The adsorption capacity, q_e_ (mg/g), was calculated according to **Equation (6)**, where C_0_ (mg/L) represents the initial metal ion concentration, C_e_ (mg/L) represents the metal ion equilibrium concentration, V (L) denotes the volume of the metal ion solution, and m (g) represents the mass of the adsorbent.

(6) $$q_{e}=\frac{\left(C_{0}-C_{e}\right)V}{m}$$

## 4. Conclusions

We developed magnetic CG–Fe_2_O_3_ NPs using *Chenopodium glaucum* L., which exhibited remarkable adsorption capabilities, particularly for selectively capturing Hg^2+^ and Pb^2+^ metal ions with maximum adsorption capacities of 96.9% and 94.1%, respectively, among various HMIs at pH 5 and with a dose of adsorbent of 0.07 g/L. The adsorption process of these metals follows the Langmuir model, indicating monolayer adsorption on the surface of CG–Fe_2_O_3_ NPs. Additionally, CG–Fe_2_O_3_ NPs serve as straightforward and highly effective green heterogeneous catalysts, achieving excellent yields in the synthesis of 2-amino-4-aryl-4H-benzo[g]chromene-3-carbonitrile derivatives. The present methodology also offers several advantages, including a straightforward experimental procedure for both catalyst preparation and product isolation, short reaction times, high yields (90–99%), tolerance towards various functional groups, and reusability, as well as the cost-effectiveness of the catalyst.

## Figures and Tables

**Table 1:** Synthesis of *2-amino-4-aryl-4H-benzo[g]chromene-3-carbonitrile* derivatives (**4a–l**) utilizing CG–Fe_2_O_3_ NPs in glycerol.

Entry	R_1_	R_f_	Isolated Yield ^a^ (%)	Melting Point (°C)	Literature Melting Point (°C)	Reference
**4a**	C_6_H_5_	0.66	95	208–209	210–211	[25]
**4b**	3-NO_2_ C_6_H_4_	0.78	97	210–211	212–214	[25]
**4c**	4-NO_2_ C_6_H_4_	0.75	95	178–179	179–182	[17]
**4d**	2-Cl C_6_H_4_	0.69	93	233–234	231–232	[6]
**4e**	4-Cl C_6_H_4_	0.70	97	230–231	231–232	[14]
**4f**	4-CH_3_ C_6_H_4_	0.58	97	203–205	202–204	[25]
**4g**	4-CH_3_O C_6_H_4_	0.63	98	192–194	192–195	[25]
**4h**	2-OH C_6_H_4_	0.71	96	245–248	253–255	[6]
**4i**	2-Thiophenyl	0.60	97	267–268	265–269	[6]
**4j**	2-Furyl	0.54	96	265–267	267–269	[17]
**4k**	4-NMe_2_ C_6_H_4_	0.78	97	209–211	210–212	[5]
**4l**	4-OH, 3-OMe C_6_H_3_	0.65	96	163–165	160–164	[6]

^a^ Yield refers to the extraction of all crops.

**Table 2:** Parameters of nonlinear Langmuir’s and Freundlich’s isotherm models using CG–Fe_2_O_3_ NPs.

Heavy Metal Ion	Langmuir’s Isotherm Model			Freundlich Isotherm Model		
	q^0^ (mg/g)	b (L/mg)	R^2^	K_F_ (mg/g)	n	R^2^
**Hg^2+^**	96.9	0.29	0.991	8.29	4.21	0.907
**Pb^2+^**	94.1	0.47	0.951	6.60	4.03	0.865

**Table 3:** Evaluation of adsorption capacity of some reported magnetic nano-adsorbents for Hg^2+^ and Pb^2+^ metal ions.

Nano-Adsorbent	Adsorption Capacity (mg/g)	Reference
Fe_3_O_4_	5.5	[39]
α-Fe_2_O_3_	11.16	[40]
Fe_2_O_3_-Ag	72.34	[15]
CL-Fe_2_O_3_	96.9–94.1	This work

## Data Availability

Data are contained within the article and Supplementary Materials.

## Supplementary Materials

The following supporting information can be downloaded at: https://www.mdpi.com/article/10.3390/molecules29194583/s1, Figure S1: Energy-Dispersive X-ray Spectroscopy (EDS) spectra of CG-Fe2O3 NPs; Figure S2: FT-IR spectra of *2-Amino-4-phenyl-4H-benzo[h]chromene-3-carbonitrile (4a)*; Figure S3: 1H-NMR spectra of *2-Amino-4-phenyl-4H-benzo[h]chromene-3-carbonitrile (4a)*; Figure S4: 13C-NMR spectra of *2-Amino-4-phenyl-4H-benzo[h]chromene-3-carbonitrile (4a)*; Figure S5: 13C-NMR expanded spectra of *2-Amino-4-phenyl-4H-benzo[h]chromene-3-carbonitrile (4a)*; Figure S6: FT-IR spectra of *2-Amino-4-(3-nitrophenyl)-4H-benzo[h]chromene-3-carbonitrile (4b)*; Figure S7: 1H-NMR spectra of *2-Amino-4-(3-nitrophenyl)-4H-benzo[h]chromene-3-carbonitrile (4b)*; Figure S8: 1H-NMR expanded spectra of *2-Amino-4-(3-nitrophenyl)-4H-benzo[h]chromene-3-carbonitrile (4b)*; Figure S9: 13C-NMR spectra of *2-Amino-4-(3-nitrophenyl)-4H-benzo[h]chromene-3-carbonitrile (4b)*; Figure S10: FT-IR spectra of *2-Amino-4-(4-nitrophenyl)-4H-benzo[h]chromene-3-carbonitrile (4c)*; Figure S11: 1H-NMR spectra of *2-Amino-4-(4-nitrophenyl)-4H-benzo[h]chromene-3-carbonitrile (4c)*; Figure S12: 1H-NMR expanded spectra of *2-Amino-4-(4-nitrophenyl)-4H-benzo[h]chromene-3-carbonitrile (4c)*; Figure S13: 13C-NMR spectra of *2-Amino-4-(4-nitrophenyl)-4H-benzo[h]chromene-3-carbonitrile (4c)*; Figure S14: 1H-NMR spectra of 2-Amino-4-(4-chlorophenyl)-4-H-benzo[g] chromene-3-carbonitrile (4e); Figure S15: 1H-NMR expanded spectra of *2-Amino-4-(4-chlorophenyl)-4-H-benzo[g] chromene-3-carbonitrile (4e)*; Figure S16: 13C-NMR spectra of *2-Amino-4-(4-chlorophenyl)-4-H-benzo[g] chromene-3-carbonitrile (4e)*; Figure S17: 13C-NMR expanded spectra of *2-Amino-4-(4-chlorophenyl)-4-H-benzo[g] chromene-3-carbonitrile (4e)*; Figure S18: FT-IR spectra of *2-Amino-4-p-tolyl-4H-benzo[h]chromene-3-carbonitrile (4f)*; Figure S19: 1H-NMR spectra of *2-Amino-4-p-tolyl-4H-benzo[h]chromene-3-carbonitrile (4f)*; Figure S20: 1H-NMR expanded spectra of *2-Amino-4-p-tolyl-4H-benzo[h]chromene-3-carbonitrile (4f)*; Figure S21: 13C-NMR spectra of *2-Amino-4-p-tolyl-4H-benzo[h]chromene-3-carbonitrile (4f)*; Figure S22: FT-IR spectra of *2-Amino-4-(4-methoxyphenyl)-4-H-benzo[h] chromene-3-carbonitrile (4g)*; Figure S23: 1H-NMR spectra of *2-Amino-4-(4-methoxyphenyl)-4-H-benzo[h] chromene-3-carbonitrile (4g)*; Figure S24: 1H-NMR expanded spectra of *2-Amino-4-(4-methoxyphenyl)-4-H-benzo[h] chromene-3-carbonitrile (4g)*; Figure S25: 13C-NMR spectra of *2-Amino-4-(4-methoxyphenyl)-4-H-benzo[h] chromene-3-carbonitrile (4g)*; Figure S26: FT-IR spectra of *2-Amino-1-(4-hydroxy-3-methoxyphenyl)-1H-benzo[f]chromene-2-carbonitrile (4l)*; Figure S27: 1H-NMR spectra of *2-Amino-1-(4-hydroxy-3-methoxyphenyl)-1H-benzo[f]chromene-2-carbonitrile (4l)*; Figure S28: 1H-NMR expanded spectra of *2-Amino-1-(4-hydroxy-3-methoxyphenyl)-1H-benzo[f]chromene-2-carbonitrile (4l)*; Figure S29: 13C-NMR spectra of *2-Amino-1-(4-hydroxy-3-methoxyphenyl)-1H-benzo[f]chromene-2-carbonitrile (4l)*; Scheme S1: Mechanism of synthesis of xanthene derivatives (4a-l) with magnetic CG-Fe2O3 NPs under MW irradiations; Scheme S2: Synthesis of xanthene derivatives (4a-l) under MW irradiations using CG-Fe3O2 NPs; Table ST1: Optimizing the reaction conditions; Table ST2: Solvent screening for the synthesis of xanthene derivative 4a; Table ST3: The influence of power on the percentage yield of 4a utilizing CG-Fe_2_O_3_ NPs in glycerol; Table ST4: Reusability of CG-Fe_2_O_3_ NPs in synthesis of xanthene 4a utilizing in glycerol; Table ST5: Synthesis of xanthene derivatives 4a-l utilizing CG-Fe_2_O_3_ NPs in glycerol; Table ST6: Adsorption values of Hg^2+^ metal ions using CG-Fe_2_O_3_ nanoparticles; Table ST7: Adsorption values of Pb^2+^ metal ions using CG-Fe_2_O_3_ nanoparticles; Table ST8: Parameters of Langmuir’s and Freundlich’s isotherm models using CG-Fe_2_O_3_ nanoparticles; Table ST9: The feasibility of the adsorption process derived from Langmuir’s isotherm model using CG-Fe_2_O_3_ nanoparticles.

## References

[1] Wang H., Lu L., Zhu S., Li Y., Cai W. (2006). The Phototoxicity of Xanthene Derivatives against *Escherichia coli*, *Staphylococcus aureus*, and *Saccharomyces cerevisiae*. Curr. Microbiol..

[2] Nikpassand M., Fekri L.Z., Ahmadi P. (2017). Grinding Synthesis of 2-Amino-4H-Chromenes Using 3,3-(butane-1,4-diyl) *bis* (1,2-dimethyl-1H-imidazole-3-ium)Br-CAN as a Novel Reagent. J. Chil. Chem. Soc..

[3] Burange A.S., Gadam K.G., Tugaonkar P.S., Thakur S.D., Soni R.K., Khan R.R., Tai M.S. (2021). Green Synthesis of Xanthene and Acridine-Based Heterocycles of Pharmaceutical Importance: A Review. Environ. Chem. Lett..

[4] Shirini F., Khaligh N.G. (2012). Succinimide-N-Sulfonic Acid: An Efficient Catalyst for the Synthesis of Xanthene Derivatives under Solvent-Free Conditions. Dye. Pigment..

[5] Banerjee A.G., Kothapalli L.P., Sharma P.A., Thomas A.B., Nanda R.K., Shrivastava S.K., Khatanglekar V.V. (2016). A Facile Microwave-Assisted One-Pot Synthesis of Novel Xanthene Derivatives as Potential Anti-Inflammatory and Analgesic Agents. Arab. J. Chem..

[6] Bosica G., De Nittis R., Borg R. (2023). Solvent-Free, One-Pot, Multicomponent Synthesis of Xanthene Derivatives. Catalysts.

[7] Nasseri M.A., Kazemnejadi M., Mahmoudi B., Assadzadeh F., Alavi S.A., Allahresani A. (2019). Efficient Preparation of 1,8-Dioxo-Octahydroxanthene Derivatives by Recyclable Cobalt-Incorporated Sulfated Zirconia (ZrO_2_/SO_4_^2−^/Co) Nanoparticles. J. Nanoparticle Res..

[8] Iniyavan P., Sarveswari S., Vijayakumar V. (2015). Microwave-Assisted Clean Synthesis of Xanthenes and Chromenes in [Bmim][PF_6_] and Their Antioxidant Studies. Res. Chem. Intermed..

[9] Sang S., Li D., Zhang H., Sun Y., Jian A., Zhang Q., Zhang W. (2017). Facile Synthesis of AgNPs on Reduced Graphene Oxide for Highly Sensitive Simultaneous Detection of Heavy Metal Ions. RSC Adv..

[10] Gao C., Yu X.-Y., Xu R.-X., Liu J.-H., Huang X.-J. (2012). AlOOH-Reduced Graphene Oxide Nanocomposites: One-Pot Hydrothermal Synthesis and Their Enhanced Electrochemical Activity for Heavy Metal Ions. ACS Appl. Mater. Interfaces.

[11] Zhao G., Tran T.-T., Modha S., Sedki M., Myung N.V., Jassby D., Mulchandani A. (2022). Multiplexed Anodic Stripping Voltammetry Detection of Heavy Metals in Water Using Nanocomposites Modified Screen-Printed Electrodes Integrated with a 3D-Printed Flow Cell. Front. Chem..

[12] Floresta G., Cardullo N., Spatafora C., Rescifina A., Tringali C. (2020). A Rare Natural Benzo[k,l]Xanthene as a Turn-off Fluorescent Sensor for Cu^2+^ Ion. Int. J. Mol. Sci..

[13] Zarei M., Zolfigol M.A., Moosavi-Zare A.R., Noroozizadeh E. (2017). Trityl Bromide versus Nano-Magnetic Catalyst in the Synthesis of Henna-Based Xanthenes and Bis-Coumarins. J. Iran. Chem. Soc..

[14] He Y., Wang Z., Ma L., Zhou L., Jiang Y., Gao J. (2020). Synthesis of Bismuth Nanoparticle-Loaded Cobalt Ferrite for Electrochemical Detection of Heavy Metal Ions. RSC Adv..

[15] Biswal S.K., Panigrahi G.K., Sahoo S.K. (2020). Green Synthesis of Fe_2_O_3_-Ag Nanocomposite Using Psidium Guajava Leaf Extract: An Eco-Friendly and Recyclable Adsorbent for Remediation of Cr(VI) from Aqueous Media. Biophys. Chem..

[16] Mannaa M.A., Mlahi M.R., AL Maofari A., Ahmed A.I., Hassan S.M. (2023). Synthesis of Highly Efficient and Recyclable Bimetallic Cox-Fe1-x-MOF for the Synthesis of Xanthan and Removal of Toxic Pb^2+^ and Cd^2+^ Ions. ACS Omega.

[17] Shirsat M.D., Hianik T. (2023). Electrochemical Detection of Heavy Metal Ions Based on Nanocomposite Materials. J. Compos. Sci..

[18] Wang C., Xu J., Zhou G., Qu Q., Yang G., Hu X. (2007). Electrochemical Detection Coupled with High-Performance Liquid Chromatography in Pharmaceutical and Biomedical Analysis: A Mini Review. Comb. Chem. High Throughput Screen..

[19] Grajeda B.A.G., Acosta S.G.S., Aguila S.A., Guevara H.P., Díaz-García M.E., Enríquez A.C., Campos-Gaxiola J.J. (2017). Selective and Colorimetric Detection of Ba^2+^ Ions in Aqueous Solutions Using 11-Mercaptoundecylphosphonic Acid Functionalized Gold Nanoparticles. RSC Adv..

[20] Amin M.A., Mersal G.A.M., El-Hendawy M.M., Shaltout A.A., Badawi A., Boman J., Gobouri A.A., Saracoglu M., Kandemirli F., Boukherroub R. (2022). Synthesis of Cyano-Benzylidene Xanthene Synthons Using a Diprotic Brønsted Acid Catalyst, and Their Application as Efficient Inhibitors of Aluminum Corrosion in Alkaline Solutions. Molecules.

[21] Miao P., Tang Y., Wang L. (2017). DNA Modified Fe_3_O_4_@Au Magnetic Nanoparticles as Selective Probes for Simultaneous Detection of Heavy Metal Ions. ACS Appl. Mater. Interfaces.

[22] Kumar A., Rout L., Achary L.S.K., Dhaka R.S., Dash P. (2017). Greener Route for Synthesis of Aryl and Alkyl-14H-Dibenzo [a.j] Xanthenes Using Graphene Oxide-Copper Ferrite Nanocomposite as a Recyclable Heterogeneous Catalyst. Sci. Rep..

[23] Sahar P., Behrooz M., Ghani M. (2024). Fe_3_O_4_@SiO_2_@Mel-Rh-Cu: A High-Performance, Green Catalyst for Efficient Xanthene Synthesis and Its Application for Magnetic Solid Phase Extraction of Diazinon Followed by Its Determination through HPLC-UV. Chem. Methodol..

[24] Fekri L.Z., Darya-Laal A.-R. (2020). NiFe_2_O_4_@SiO_2_@amino Glucose Magnetic Nanoparticle as a Green, Effective and Magnetically Separable Catalyst for the Synthesis of Xanthene-Ones under Solvent-Free Condition. Polycycl. Aromat. Compd..

[25] Mousavi S.R., Nodeh H.R., Afshari E.Z., Foroumadi A. (2019). Graphene Oxide Incorporated Strontium Nanoparticles as a Highly Efficient and Green Acid Catalyst for One-Pot Synthesis of Tetramethyl-9-Aryl-Hexahydroxanthenes and 13-Aryl-5H-Dibenzo[b,i]Xanthene-5,7,12,14(13H)-Tetraones Under Solvent-Free Conditions. Catal. Lett..

[26] El-Yazeed W.S.A., Hayes O.R., Ahmed A.I. (2021). Phosphotungestic Acid Supported Mesoporous MCM-41 Coated NiFe_2_O_4_ Magnetic Nanoparticles as Highly Effective Green Nanocatalysts for Coumarin and Xanthene Synthesis. J. Sol-Gel Sci. Technol..

[27] Haeri H.S., Rezayati S., Nezhad E.R., Darvishi H. (2016). Fe^2+^ Supported on Hydroxyapatite-Core–Shell-γ-Fe_2_O_3_ Nanoparticles: Efficient and Recyclable Green Catalyst for the Synthesis of 14-Aryl-14H-Dibenzo[a,j]Xanthene Derivatives. Res. Chem. Intermed..

[28] Liu L., Yuan M., Huang S., Li J., Li D., Zhao L. (2018). Analysis of Xanthine Oxidase Inhibitors from *Clerodendranthus spicatus* with Xanthine Oxidase Immobilized Silica Coated Fe_3_O_4_ Nanoparticles. Appl. Sci..

[29] Barman G., Samanta A., Maiti S., Laha J.K. (2014). Colorimetric Assays for the Detection of Hg(II) Ions Using Functionalized Gold and Silver Nanoparticles. Adv. Sci. Focus.

[30] Rostamizadeh E., Iranbakhsh A., Majd A., Arbabian S., Mehregan I. (2020). Green Synthesis of Fe_2_O_3_ Nanoparticles Using Fruit Extract of *Cornus mas* L. and Its Growth-Promoting Roles in Barley. J. Nanostruct. Chem..

[31] Shankramma K., Yallappa S., Shivanna M.B., Manjanna J. (2016). Fe_2_O_3_ Magnetic Nanoparticles to Enhance *S. lycopersicum* (Tomato) Plant Growth and Their Biomineralization. Appl. Nanosci..

[32] Ahmad W., Joshi H.C., Pandey S., Kumar V., Verma M. (2023). An Overview of Green Methods for Fe_2_O_3_ Nanoparticle Synthesis and Their Applications. Int. Nano Lett..

[33] Campos E.A., Pinto D.V.B.S., de Oliveira J.I.S., Mattos E.d.C., Dutra R.d.C.L. (2015). Synthesis, Characterization and Applications of Iron Oxide Nanoparticles—A Short Review. Int. J. Health Sci..

[34] Karpagavinayagam P., Vedhi C. (2019). Green Synthesis of Iron Oxide Nanoparticles Using Avicennia Marina Flower Extract. Vacuum.

[35] Waseem M., Munsif S., Rashid U. (2014). Imad-ud-Din Physical Properties of α-Fe_2_O_3_ Nanoparticles Fabricated by Modified Hydrolysis Technique. Appl. Nanosci..

[36] Bhosale M.A., Ummineni D., Sasaki T., Nishio-Hamane D., Bhanage B.M. (2015). Magnetically Separable γ-Fe_2_O_3_ Nanoparticles: An Efficient Catalyst for Acylation of Alcohols, Phenols, and Amines Using Sonication Energy under Solvent Free Condition. J. Mol. Catal. A Chem..

[37] Powell C.D., Lounsbury A.W., Fishman Z.S., Coonrod C.L., Gallagher M.J., Villagran D., Zimmerman J.B., Pfefferle L.D., Wong M.S. (2021). Nano-Structural Effects on Hematite (α-Fe_2_O_3_) Nanoparticle Radiofrequency Heating. Nano Converg..

[38] Mollie E., Annette S., Butler J. (1991). The Atom Economy A Search for Synthetic Efficiency. Green Chem. Teach. Learn. Community.

[39] Luo H., Zhang S., Li X., Liu X., Xu Q., Liu J., Wang Z. (2017). Tannic acid modified Fe_3_O_4_ core–shell nanoparticles for adsorption of Pb^2+^ and Hg^2+^. J. Taiwan Inst. Chem. Eng..

[40] Zhang Z., Liu H., Lu P., Chen T., Ma W. (2018). Nanostructured α-Fe_2_O_3_ Derived from Siderite as an Effective Hg(II) Adsorbent: Performance and Mechanism. Appl. Geochem..

