# Supplementary material for: Green Synthesis of Magnetic Fe2O3 Nanoparticle with Chenopodium glaucum L. as Recyclable Heterogeneous Catalyst for One-Pot Reactions and Heavy Metal Adsorption
Source: Molecules. 2024 Sep 26;29(19):4583. doi: 10.3390/molecules29194583 (PMC11478018; doi:10.3390/molecules29194583)
Supplement: Supplementary file 1 [file molecules-29-04583-s001.zip › molecules-3168485-supplementary.pdf]

Electronic Supplementary Information

Green Synthesis of Magnetic Fe_2O_3 Nanoparticle with *Chenopodium glaucum* L. as Recyclable Heterogeneous Catalyst for One-Pot Reactions and Heavy Metal Adsorption

Rahul Thakur [1], Navneet Kaur [1], Manvinder Kaur [1], Pradip K. Bhowmik [2], Haesook Han [2], Kishanpal Singh [3], Fohad Mabood Husain [4] and Harvinder Singh Sohal [1,*]

[1] Medicinal and Natural Product Laboratory, Department of Chemistry, Chandigarh University, Gharuan, Mohali 140413, Punjab, India
[2] Department of Chemistry and Biochemistry, University of Nevada Las Vegas, 4505 S. Maryland Parkway, Box 454003, Las Vegas, NV 89154, USA
[3] Department of Chemistry, Punjabi University, Patiala 147002, Punjab, India
[4] Department of Food Science and Nutrition, College of Food and Agriculture Sciences, King Saud University, Riyadh 11451, Saudi Arabia
* Correspondence: drharvinder.cu@gmail.com

1. Results and Discussion

1.1. Preparation of 2-amino-4-aryl-4H-benzo[g]chromene-3-carbonitrile derivatives (4a-l) using magnetic CG-Fe_2O_3 nanoparticles

The catalyst screening was carried out to identify the optimal concentration of CG-Fe_2O_3 NPs for the reactions to occur with higher efficiency. Experimental results, as shown in **Table ST1**, show varying CG-Fe_2O_3 NPs concentrations affect reaction time and isolated yield. Optimal concentrations obtained higher yields: 92% at 5 and 7 mmol% concentration in 9 and 8 minutes, respectively. The increase in the quantity of catalyst exhibits minimal impact on the yield percentage within the same timeframe which indicates that 7 mmol% is an optimal range of CG-Fe_2O_3 NPs concentration for efficient reactions.

Table ST2. Optimizing the reaction conditions.

Fe_2O_3-NPs (mmol%)	Time (min.)	Isolated Yield[a,*] (%)
0	6	55%
3	5	76%
5	9	92%
**7**	**8**	**92%**
9	8	93%

[a]Yield refers to extraction of all crops. *Reaction conditions: *β*-naphthol (1 eq.), benzaldehyde (1 eq.), malononitrile (1 eq.) at 100 watts.

According to the screening of different solvents for the condensation reaction which involves *β*-naphthol **1** (5 mmol), benzaldehyde **2a** (5 mmol), along with malononitrile **3** (5 mmol) using CG-Fe_2O_3 NPs (7 mmol%) at 100 watts for 8 minutes, glycerol emerged as the most efficient solvent which gives higher yield of xanthene in shorter time as compared to others as shown in **Table ST2**.

Table ST2. Solvent screening for the synthesis of xanthene derivative **4a**.

Solvent	Time (min.)	Isolated Yield[a,*] (%)
Ethanol	9	92%
Methanol	11	82%
Water	15	35%
Acetonitrile	14	70%
**Glycerol**	**7**	**95%**
Polyethylene glycol	20	75%
Ethylene glycol	17	77%

[a]Yield refers to extraction of all crops. *Reaction conditions: *β*-naphthol (1 eq.), benzaldehyde (1 eq.), malononitrile (1 eq.), and CG-Fe_2O_3 NPs (7 mmol%) at 100 watts.

In **Table ST3**, we discussed the optimization of the reaction power for a reaction performed in glycerol using CG-Fe_2O_3 NPs. It was observed that as the power increased, there was a significant rise in yield over time. However, excessive power elevation resulted in a decrease in product yield due to product decomposition at higher power.

Table ST3. The influence of power on the percentage yield of **4a** utilizing CG-Fe_2O_3 NPs in glycerol.

Watt	Time (min.)	Isolated Yield[a,*] (%)
90	12	87%
100	7	95%
110	7	93%
**120**	**6**	**97%**
130	6	96%

[a]Yield refers to extraction of all crops. *Reaction conditions: β-naphthol (1 eq.), benzaldehyde (1 eq.), malononitrile (1 eq.), and CG-Fe_2O_3 NPs (7 mmol%).

Using the optimized conditions, we examined the reusability of CG-Fe_2O_3 NPs in the reaction. After filtration from the previous reaction, the nanoparticles were washed with dry ethyl acetate and vacuum dried before reuse for xanthene derivative synthesis. Remarkably, product **4a** was obtained with a high yield of 97%. The catalyst retained good activity over three runs, with no notable decrease in yield observed **(Table ST4)**, confirming its continued effectiveness.

Table ST4. Reusability of CG-Fe_2O_3 NPs in synthesis of xanthene **4a** utilizing in glycerol.

Run	Isolate Yield[a,*] (%)
Fresh	97%
1	96%
2	94%

[a]Yield refers to extraction of all crops. *Reaction conditions: β-naphthol (1 eq.), benzaldehyde (1 eq.), malononitrile (1 eq.), and CG-Fe_2O_3 NPs (7 mmol%) at 120 watts for 6 min.

1.2. Plausible Mechanism

As per the literature, the proposed mechanism as shown in **Scheme S1** involves Knoevenagel condensation between substituted-benzaldehyde **2** and malononitrile **3**, followed by Michael addition with β-naphthol **1** to form a dihydrochromene intermediate. Tautomerization and intramolecular cyclization then yield the final chromene product through consecutive steps of deprotonation, nucleophilic attack, tautomerization, and ring closure.

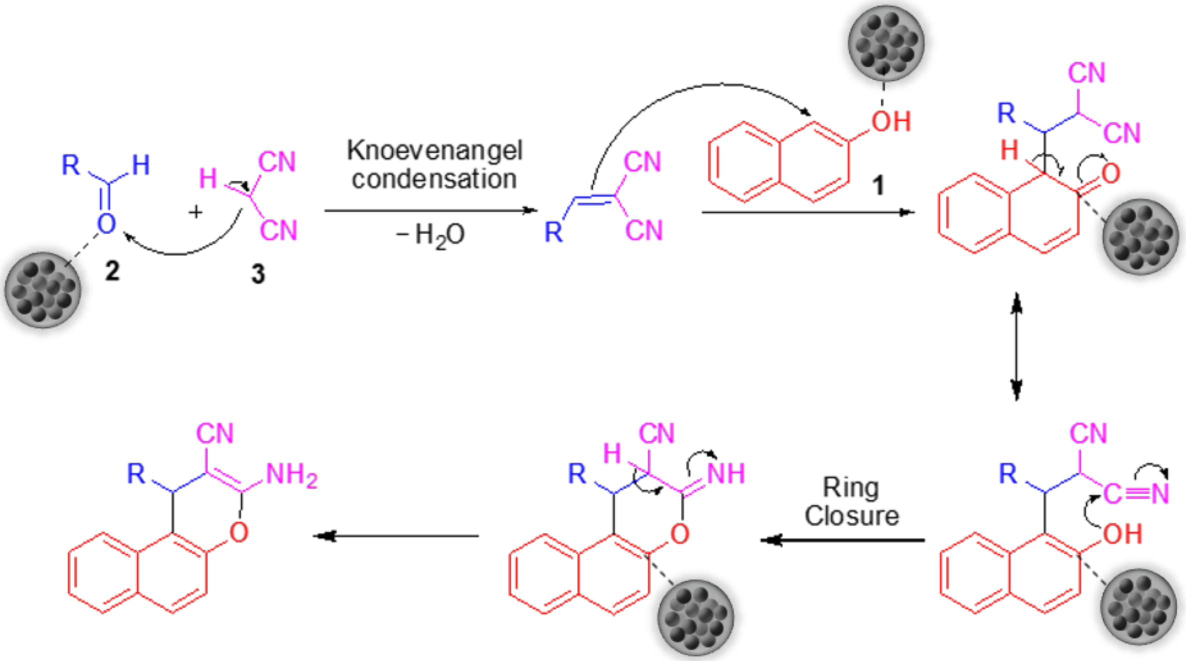

Scheme S1. Mechanism of synthesis of xanthene derivatives **(4a-l)** with magnetic CG-Fe_2O_3 NPs under MW irradiations.

2. Materials and Methods

2.1. Chemicals

All chemicals utilized in this study were procured from Sigma-Aldrich (Punjab, India) and were employed without further distillation. Solvents were sourced from Loba Chemie (Punjab, India).

2.2. Analytical instruments

The melting points of synthesized products were determined using a digital melting point apparatus *via* the open capillary method. FT-Infrared (FT-IR) spectra were obtained using the attenuated total reflection (ATR) mode on a Perkin Elmer Spectrum II instrument. 1H-NMR and 13C-NMR spectra were recorded on a Bruker Avance NEO 500 MHz FT-NMR instrument with $CDCl_3$ as the solvent, at 300 and 400 MHz, respectively, referencing chemical shifts (δ) to tetramethyl silane (TMS) as an internal standard. The thin-layer chromatography (TLC) technique, coupled with visualization in a UV chamber, monitored reaction progress and assessed compound purity. The magnetic characteristics of the adsorbents were assessed using a vibrating sample magnetometer (VSM, 7410 Series, Lake Shore). The zero point charge (ZPC) and the hydrodynamic dimensions of the particles were measured using a Zetasizer Nano-ZS90 instrument (Chandigarh University,

India). Metal ion concentrations were determined using an inductively coupled plasma optical emission spectrometer (ICP-OES, 5110 ICP-OES, Agilent Technologies, PAU, Ludhiana).

2.3. Synthesis of CG-Fe_2O_3 Nanoparticles

Combine 0.4 M ferric chloride and 0.2 M ferrous sulfate solution under nitrogen atmosphere, stirred for 15 minutes at 70 °C. Then slowly added *C. glaucum* until pH 11. Filtered precipitates, washed with acetone and water until pH 7. Dried at 70 °C for 24 hrs, then calcinated at 500 °C for 5 hrs to obtain CG-Fe_2O_3 NPs.

2.4. Synthesis of xanthene derivatives (4a-l)

A mixture composed of β-naphthol **1** (5 mmol), substituted benzaldehyde **2** (5 mmol), along with malononitrile **3** (5 mmol), in the presence of CG-Fe_2O_3 NPs (7 mmol%) was subjected to MW irradiation at 120 watt for 6 minutes in glycerol (5 mL) **(Scheme S2)**. After completion of the reaction, the mixture was cooled to room temperature, recover magnetic nanoparticles *via* magnet, filtered off the solution, then washed with distilled water, and subsequently, the resulting solid was recrystallized using ethanol to afford the pure crystals with an efficiency of 90-95%. All reactions were monitored using TLC with silica gel-coated plates (ethyl acetate: hexane/ 6:4/ v:v).

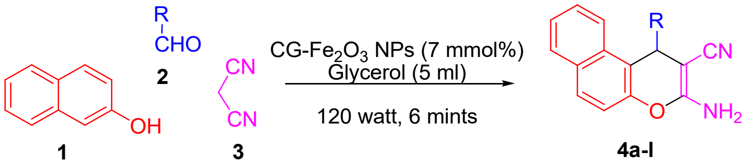

Scheme S2. Synthesis of xanthene derivatives **(4a-l)** under MW irradiations using CG-Fe_2O_3 NPs.

In a similar manner, different aldehydes **2b-l** will undergo reaction with *β*-naphthol **1**, and malononitrile **3** using the same protocol. The progress of the reactions will be monitored through TLC and melting point analysis **(Table ST5)**. These reactions proceed smoothly, even in the presence of various electron-donating (-Me and -OMe) as well as electron-withdrawing substituents (-NO_2) on the aldehyde, facilitating efficient reactions. The effectiveness of this approach is remarkable, yielding high percentages (92-97%) for the final products.

Table ST5. Synthesis of xanthene derivatives **4a-l** utilizing CG-Fe_2O_3 NPs in glycerol.

Entry	R_1	Rf	Isolated Yield[a] (%)	Melting Point (°C)	Literature Melting Point (°C)	Reference
**4a**	C_6H_5	0.66	95	208-209	210-211	[25]
**4b**	3-NO_2 C_6H_4	0.78	97	210-211	212-214	[25]
**4c**	4-NO_2 C_6H_4	0.75	95	178-179	179-182	[17]
**4d**	2-Cl C_6H_4	0.69	93	233-234	231-232	[6]
**4e**	4-Cl C_6H_4	0.70	97	230-231	231-232	[14]
**4f**	4-CH_3 C_6H_4	0.58	97	203-205	202-204	[25]
**4g**	4-CH_3O C_6H_4	0.63	98	192-194	192-195	[25]
**4h**	2-OH C_6H_4	0.71	96	245-248	253-255	[6]
**4i**	2-Thiophenyl	0.60	97	267-268	265-269	[6]
**4j**	2-Furyl	0.54	96	265-267	267-269	[17]
**4k**	4-NMe_2 C_6H_4	0.78	97	209-211	210-212	[5]
**4l**	4-OH, 3-OMe C_6H_3	0.65	96	163-165	160-164	[6]

[a]Yield refers to extraction of all crops.

2.5. Isotherm studies

Table ST6. Adsorption values of Hg^{2+} metal ions using CG-Fe_2O_3 nanoparticles.

S. No.	C_i (mg/l)	C_e (mg/l)	log C_e	ln C_e	q_e (mg/g)
1	10	3.1	0.491	1.131	9.857
2	30	8.6	0.934	2.152	30.571
3	50	15.2	1.182	2.721	49.714
4	100	41.3	1.616	3.721	83.857
5	250	151.8	2.181	5.023	140.286
6	500	391.7	2.593	5.970	154.714

Table ST7. Adsorption values of Pb^{2+} metal ions using CG-Fe_2O_3 nanoparticles.

S. No.	C_i (mg/L)	C_e (mg/L)	log C_e	ln C_e	q_e (mg/g)
1	10	4.2	0.623	1.435	8.286
2	30	9.2	0.964	2.219	29.714

3	50	17.5	1.243	2.862	46.429
4	100	43.2	1.635	3.766	81.143
5	250	160.8	2.206	5.080	127.429
6	500	401.3	2.603	5.995	141.000

Table ST8. Parameters of Langmuir's and Freundlich's isotherm models using CG-Fe_2O_3 nanoparticles.

Heavy metal ion	Langmuir's isotherm model			Freundlich isotherm model		
	**Slope**	**intercept**	**R^2**	**Slope**	**intercept**	**R^2**
**Hg^{2+}**	0.297	0.003	0.992	0.237	0.919	0.907
**Pb^{2+}**	0.472	-0.0005	0.951	0.248	0.821	0.865

Table ST9. The feasibility of the adsorption process derived from Langmuir's isotherm model using CG-Fe_2O_3 nanoparticles.

**C_o (mg/L)**	**Hg^{2+}; b = 0.29**	**Pb^{2+}; b = 0.47**
	**R_L**	**R_L**
10	0.256	0.175
30	0.103	0.066
50	0.065	0.041
100	0.033	0.021
250	0.014	0.009
500	0.007	0.004

2.6. Characterization of synthesized xanthene derivatives

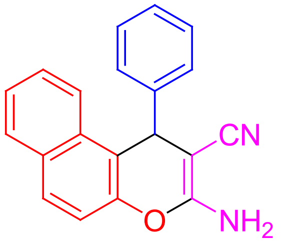

4a

2-Amino-4-phenyl-4H-benzo[h]chromene-3-carbonitrile ***(4a):*** Yield 95%, colourless crystals, MP 208-209 ºC; Lit. MP (210-211 °C). FT-IR spectrum, υ, cm^{-1}: 2182.36 (C≡N), 3338.00 (N-H), 3432.74 (sp^2 hybridized C-H bonds), 1589.45 (C=C aromatic stretching vibrations). ^{1}H-NMR spectrum, δ, ppm (*J*, Hz): 7.14-7.95 (Ar-H), 5.30 (*s*, 1H, CH), 6.98 (*s*, 1H, NH_2). ^{13}C-NMR

spectrum, δ, ppm: 159.61, 146.74, 145.60, 130.73, 130.07, 129.39, 128.60, 128.36, 126.97, 126.89, 126.50, 124.83, 123.52, 121.37, 120.39, 116.69, 115.58, 57.83, 38.00.

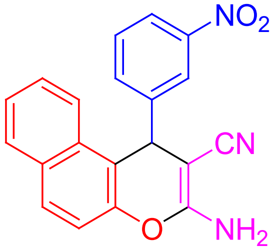

4b

2-Amino-4-(3-nitrophenyl)-4H-benzo[h]chromene-3-carbonitrile ***(4b):*** Yield 97%, colourless crystals, MP 210-211 ºC; Lit. MP (212-214 °C). FT-IR spectrum, υ, cm^{-1}: 2190.31 (C≡N), 3353.48 (N-H), 3463.45 (sp^2 hybridized C-H bonds), 1589.63 (C=C aromatic stretching vibrations). ^{1}H-NMR spectrum, δ, ppm (*J*, Hz): 7.37-8.10 (Ar-H), 5.62 (*s*, 1H, CH), 7.18 (*s*, 1H, NH_2). ^{13}C-NMR spectrum, δ, ppm: 159.92, 147.90, 147.76, 146.90, 133.58, 130.76, 130.28, 129.94, 129.83, 128.47, 127.27, 125.01, 123.35, 121.69, 120.11, 116.74, 114.47, 56.98.

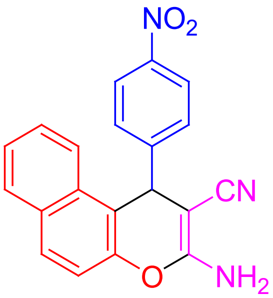

4c

2-Amino-4-(4-nitrophenyl)-4H-benzo[h]chromene-3-carbonitrile ***(4c):*** Yield 97%, colorless crystals, MP 178-179 ºC; Lit. MP (179-182 °C). FT-IR spectrum, υ, cm^{-1}: 2190.14 (C≡N), 3331.02 (N-H), 3426.62 (sp^2 hybridized C-H bonds), 1588.39 (C=C aromatic stretching vibrations). ^{1}H-NMR spectrum, δ, ppm (*J*, Hz): 7.36-8.14 (Ar-H), 5.55 (*s*, 1H, CH), 7.15 (*s*, 1H, NH_2). ^{13}C-NMR spectrum, δ, ppm: 159.81, 152.82, 146.80, 146.08, 130.73, 129.92, 129.87, 128.46, 128.12, 127.22, 124.99, 123.99, 123.28, 121.24, 119.99, 116.74, 114.32, 56.51.

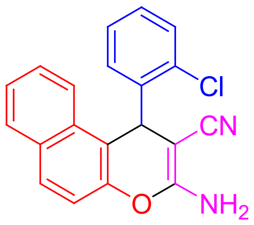

4d

2-Amino-4-(2-chlorophenyl)-4-H-benzo[g] chromene-3-carbonitrile ***(4d):*** Yield 93.6%, colorless crystals, MP 233-234 ºC; Lit. MP (231-232 °C). FT-IR spectrum, υ, cm^{-1}: 3173.28 (C≡N), 3298.17 (N-H), 3419.28 (sp^2 hybridized C-H bonds), 1568.91 (C=C aromatic stretching vibrations). ^{1}H-NMR (250 MHz, DMSO-d6) δ (ppm): 7.22-8.22 (Ar-H), 5.38 (*s*, 1H, CH), 6.96 (*s*, 1H, NH_2). ^{13}C-NMR (62.9 MHz, DMSO-d6) δ (ppm): 160.22, 152.08, 146.71, 146.46, 130.66, 129.64, 129.33, 128.94, 128.85, 127.70, 124.44, 123.96, 123.02, 120.18, 119.66, 116.27, 114.12, 56.56.

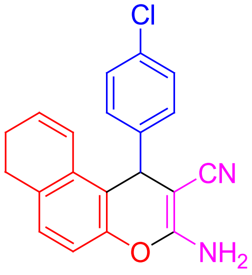

4e

2-Amino-4-(4-chlorophenyl)-4-H-benzo[g] chromene-3-carbonitrile ***(4e):*** Yield 97.9%, Yellow crystals, MP 230-231 ºC; Lit. MP (231-232 °C). FT-IR spectrum, υ, cm^{-1}: 3466.53 (C≡N), 3328.22 (N-H), 3431.81 (sp^2 hybridized C-H bonds), 1577.14 (C=C aromatic stretching vibrations). ^{1}H-NMR (250 MHz, DMSO-d6) δ (ppm): 7.08-7.97 (Ar-H), 7.08 (*s*, 1H, CH), 5.36 (*s*, 1H, NH_2). ^{13}C-NMR (62.9 MHz, DMSO-d6) δ (ppm): 159.79, 146.85, 144.62, 131.28, 130.86, 130.07, 129.73, 128.84, 128.71, 128.54, 127.21, 123.50, 120.43, 116.86, 115.08, 57.54, 39.92, 37.51.

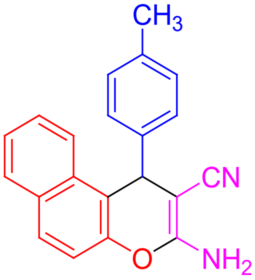

4f

2-Amino-4-p-tolyl-4H-benzo[h]chromene-3-carbonitrile ***(4f):*** Yield 97%, colorless crystals, MP 203-205 ºC; Lit. MP (202-204 °C). FT-IR spectrum, υ, cm^{-1}: 2187.77 (C≡N), 3336.16 (N-H), 3426.95 (sp^2 hybridized C-H bonds), 1591.27 (C=C aromatic stretching vibrations). ^{1}H-NMR spectrum, δ, ppm (*J*, Hz): 7.04-7.93 (Ar-H), 5.25 (*s*, 1H, CH), 6.95 (*s*, 1H, NH_2). ^{13}C-NMR spectrum, δ, ppm: 159.50, 146.65, 142.69, 135.56, 130.70, 130.08, 129.27, 129.11, 128.31, 126.89, 126.77, 124.75, 123.53, 120.42, 119.56, 116.65, 115.67, 57.98, 37.56.

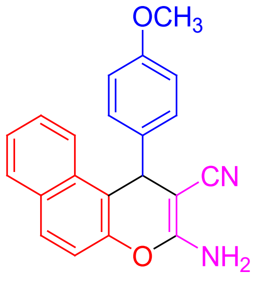

4g

2-Amino-4-(4-methoxyphenyl)-4-H-benzo[h] chromene-3-carbonitrile ***(4g):*** Yield 98%, yellow crystals, MP 192-194 ºC; Lit. MP (192-195 °C). FT-IR spectrum, υ, cm^{-1}: 2188.25 (C≡N), 3343.94 (N-H), 3430.57 (sp^2 hybridized C-H bonds), 1588.73 (C=C aromatic stretching vibrations). ^{1}H-NMR spectrum, δ, ppm (*J*, Hz): 6.76-7.79 (Ar-H), 4.61 (*s*, 1H, CH), 5.18 (*s*, 1H, NH_2). ^{13}C-NMR spectrum, δ, ppm: 158.52, 158.46, 147.04, 136.76, 131.41, 130.79, 129.52, 128.47, 128.21, 127.17, 125.07, 120.01, 120.37, 116.65, 115.23, 114.29, 62.56, 55.19.

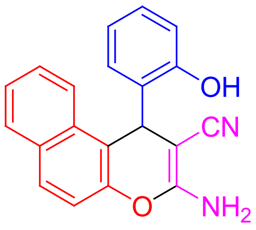

4h

2-Amino-4-(2-hydroxyphenyl)-4-H-benzo[h] chromene-3-carbonitrile ***(4h):*** Yield 90%, Yellow crystals, MP 245-248 ºC; Lit. MP (253-255 °C). FT-IR spectrum, υ, cm^{-1}: 3264.93 (C≡N), 3333.15 (N-H), 3415.64 (sp^2 hybridized C-H bonds), 1579.96 (C=C aromatic stretching vibrations). ^{1}HNMR (500 MHz, DMSO-d6) δ (ppm): 6.99-8.20 (Ar-H), 4.74 (*s*, 1H, CH), 5.21 (*s*, 1H, NH_2). ^{13}C-NMR (62.9 MHz, DMSO-d6) δ (ppm): 157.92, 158.15, 147.24, 136.13, 131.25, 130.55, 129.10, 128.34, 128.45, 127.38, 125.87, 123.81, 120.52, 116.12, 115.24, 114.87, 55.26.

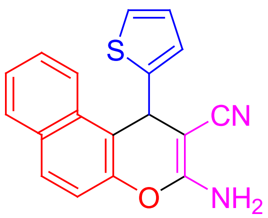

4i

2-Amino-4-(thiophen-2-yl)-4-H-benzo[h]chromene3-carbonitrile ***(4i):*** Yield 97%, yellow crystals, MP 267-268 ºC; Lit. MP (265-269 °C). FT-IR spectrum, υ, cm^{-1}: 3187.81 (C≡N), 3316.27

(N-H), 3465.18 (sp^2 hybridized C-H bonds), 1597.38 (C=C aromatic stretching vibrations). ^{1}HNMR (500 MHz, DMSO-d6) δ (ppm): 6.88-8.19 (Ar-H), 4.99 (*s*, 1H, CH), 5.37 (*s*, 1H, NH_2). ^{13}C-NMR (62.9 MHz, DMSO-d6) δ (ppm): 156.82, 157.93, 146.91, 135.90, 130.86, 130.37, 130.07, 127.15, 127.93, 127.11, 125.53, 123.81, 120.17, 115.94, 115.13, 114.72, 55.15.

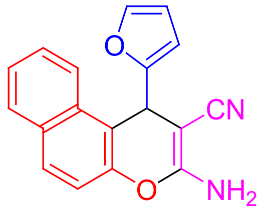

4j

2-Amino-4-(furan-2-yl)-4-H-benzo[h]chromene3-carbonitrile ***(4j):*** Yield 96%, yellow crystals, MP 265-267 ºC; Lit. MP (267-269 °C). FT-IR spectrum, ʋ, cm^{-1}: 3227.51 (C≡N), 3327.07 (N-H), 3417.26 (sp^2 hybridized C-H bonds), 1583.18 (C=C aromatic stretching vibrations). ^{1}HNMR (500 MHz, DMSO-d6) δ (ppm): 6.27-7.89 (Ar-H), 5.19 (*s*, 1H, CH), 5.27 (*s*, 1H, NH_2). ^{13}C-NMR (62.9 MHz, DMSO-d6) δ (ppm): 157.02, 158.23, 147.61, 136.83, 129.99, 130.27, 130.19, 128.29, 128.13, 127.38, 125.09, 123.88, 116.04, 116.73, 114.37, 55.15.

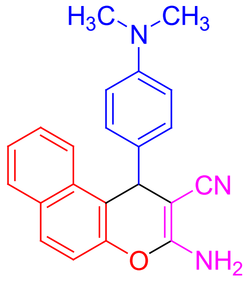

4k

2-amino-1-(4-(dimethylamino)phenyl)-4-H-benzo[h]chromene—carbonitrile ***(4k):*** Yield 97%, white crystals, MP 209-211 ºC; Lit. MP (210-212 °C). FT-IR spectrum, ʋ, cm^{-1}: 3465.72 (C≡N), 3318.37 (N-H), 3437.43 (sp^2 hybridized C-H bonds), 1585.37 (C=C aromatic stretching vibrations). ^{1}HNMR (500 MHz, DMSO-d6) δ (ppm): 7.67-7.17 (Ar-H), 5.76 (*s*, 1H, CH), 5.12 (*s*, 1H, NH_2). ^{13}C-NMR (62.9 MHz, DMSO-d6) δ (ppm): 158.52, 153.40, 148.04, 146.34, 143.95, 138.16, 134.78, 133.46, 132.33, 130.54, 129.68, 128.54, 127.02, 124.42, 123.03, 122.43, 122.43, 121.94, 119.03, 67.54, 56.93, 44.83.

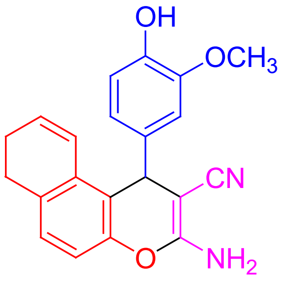

4l

2-Amino-1-(4-hydroxy-3-methoxyphenyl)-1H-benzo[f]chromene-2-carbonitrile ***(4l):*** Yield 96%, yellow crystals, MP 163-165 ºC; Lit. MP (160-164 °C) FT-IR spectrum, υ, cm^{-1}: 2218.35 (C≡N), 3127.74 (N-H), 3421.67 (sp^2 hybridized C-H bonds), 3362.14 (O-H bending), 1585.85 (C=C aromatic stretching vibrations). ^{1}H-NMR spectrum, δ, ppm (*J*, Hz): 6.40-7.76 (Ar-H), 5.24 (*s*, 1H, CH), 6.40 (*s*, 1H, NH_2), 3.95 (s, 1H, OCH_3). ^{13}C-NMR spectrum, δ, ppm: 159.34, 153.42, 152.17, 147.07, 134.61, 129.83, 129.03, 128.93, 127.77, 126.52, 126.37, 124.00, 123.60, 117.78, 115.24, 114.42, 113.64, 110.46, 109.50, 56.22.

3. SEM Characterization

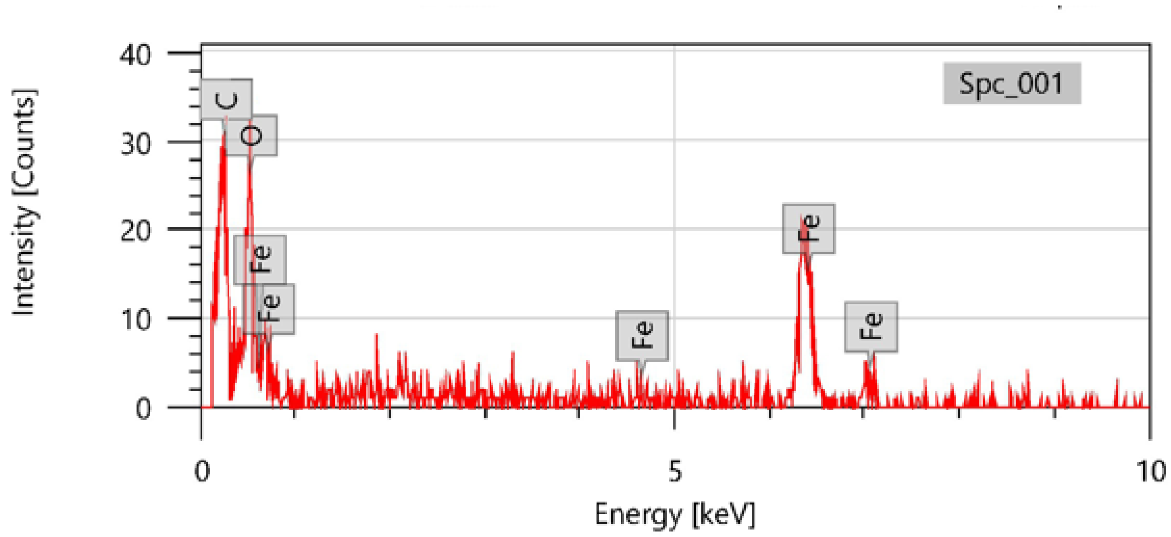

Figure S1. Energy-Dispersive X-ray Spectroscopy (EDS) spectra of CG-Fe_2O_3 NPs.

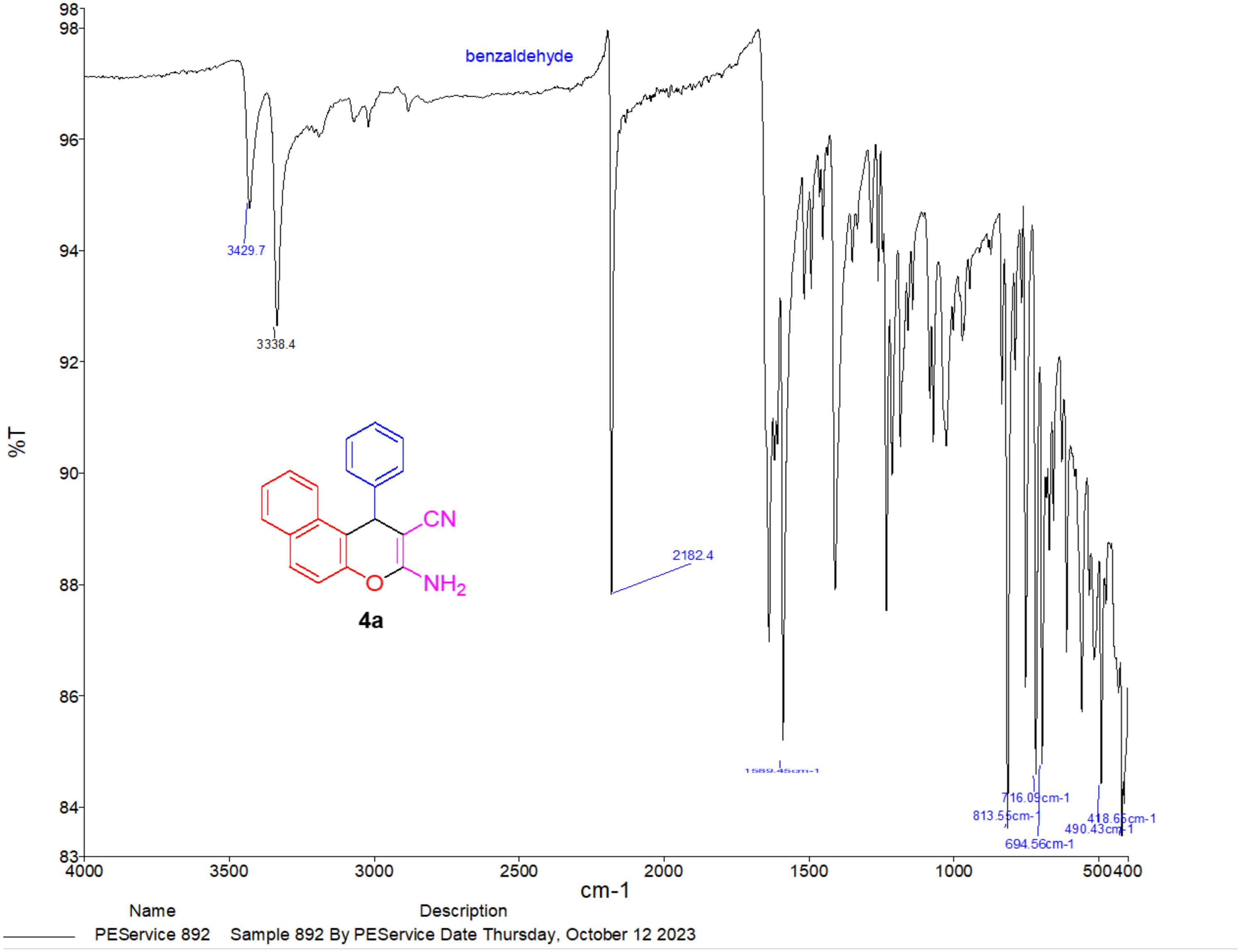

Figure S2. FT-IR spectrum of *2-Amino-4-phenyl-4H-benzo[h]chromene-3-carbonitrile* ***(4a)***.

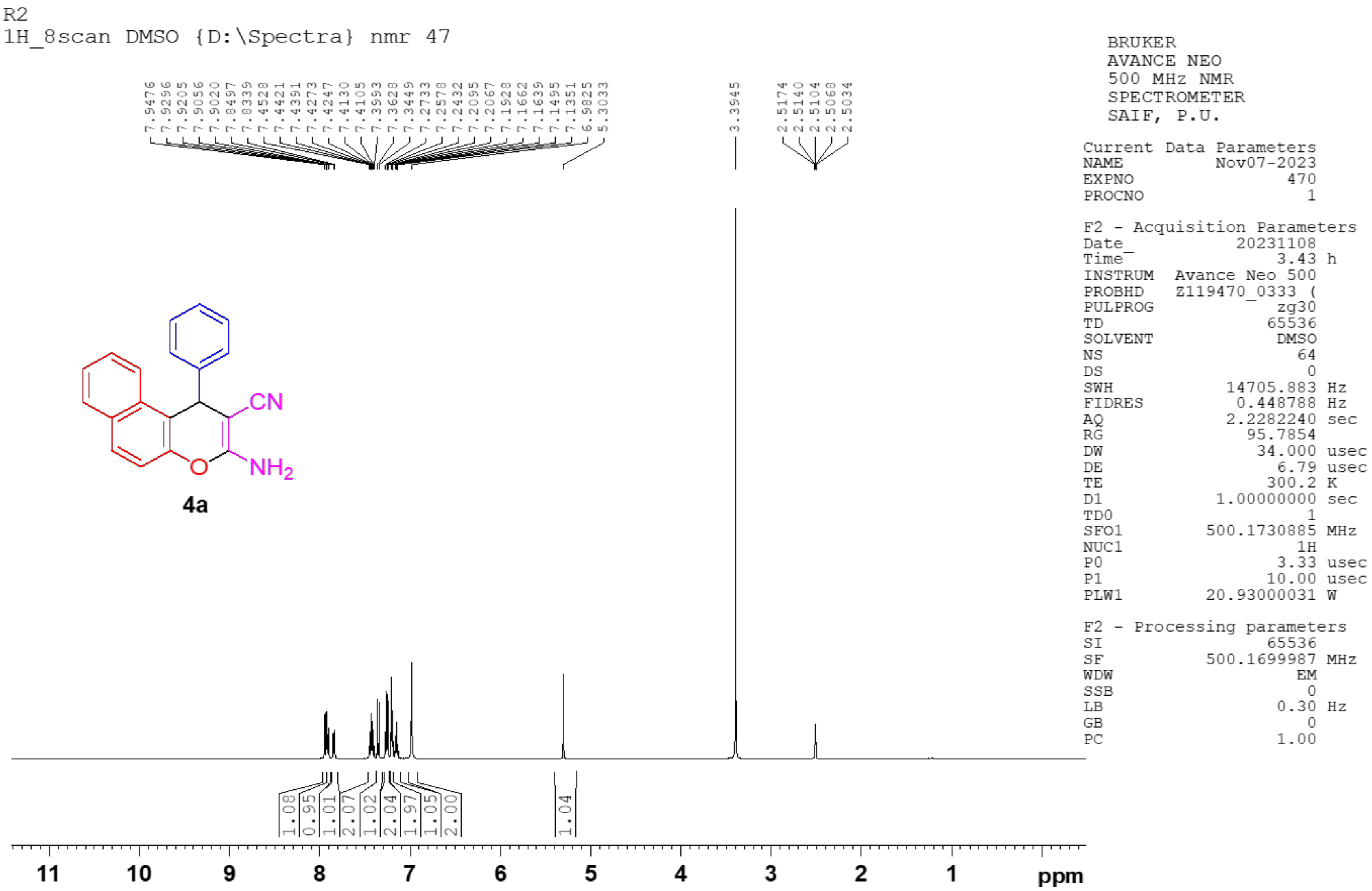

Figure S3. ¹H-NMR spectrum of *2-Amino-4-phenyl-4H-benzo[h]chromene-3-carbonitrile* ***(4a)***.

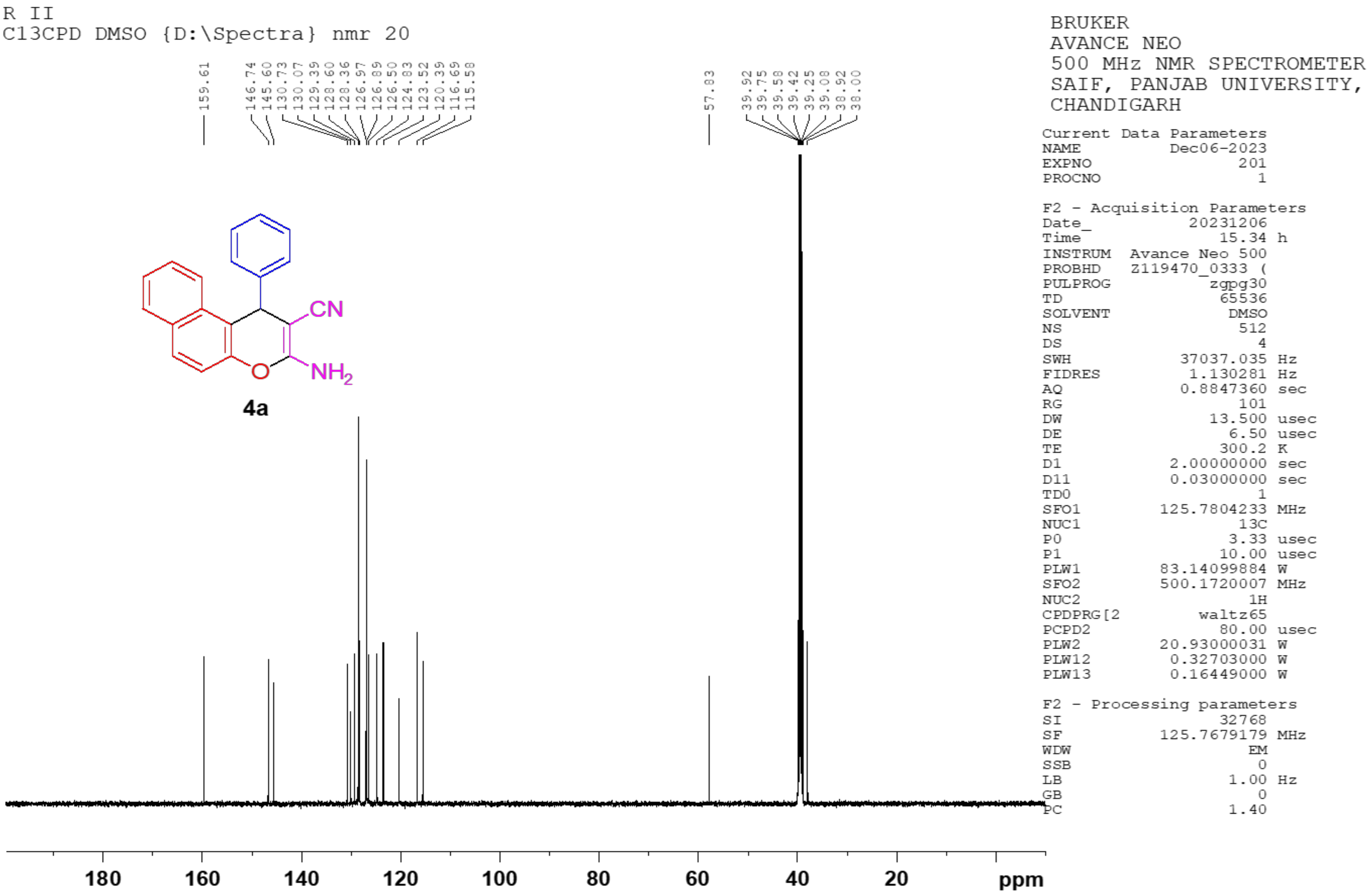

Figure S4. ^{13}C-NMR spectrum of *2-Amino-4-phenyl-4H-benzo[h]chromene-3-carbonitrile* ***(4a)***.

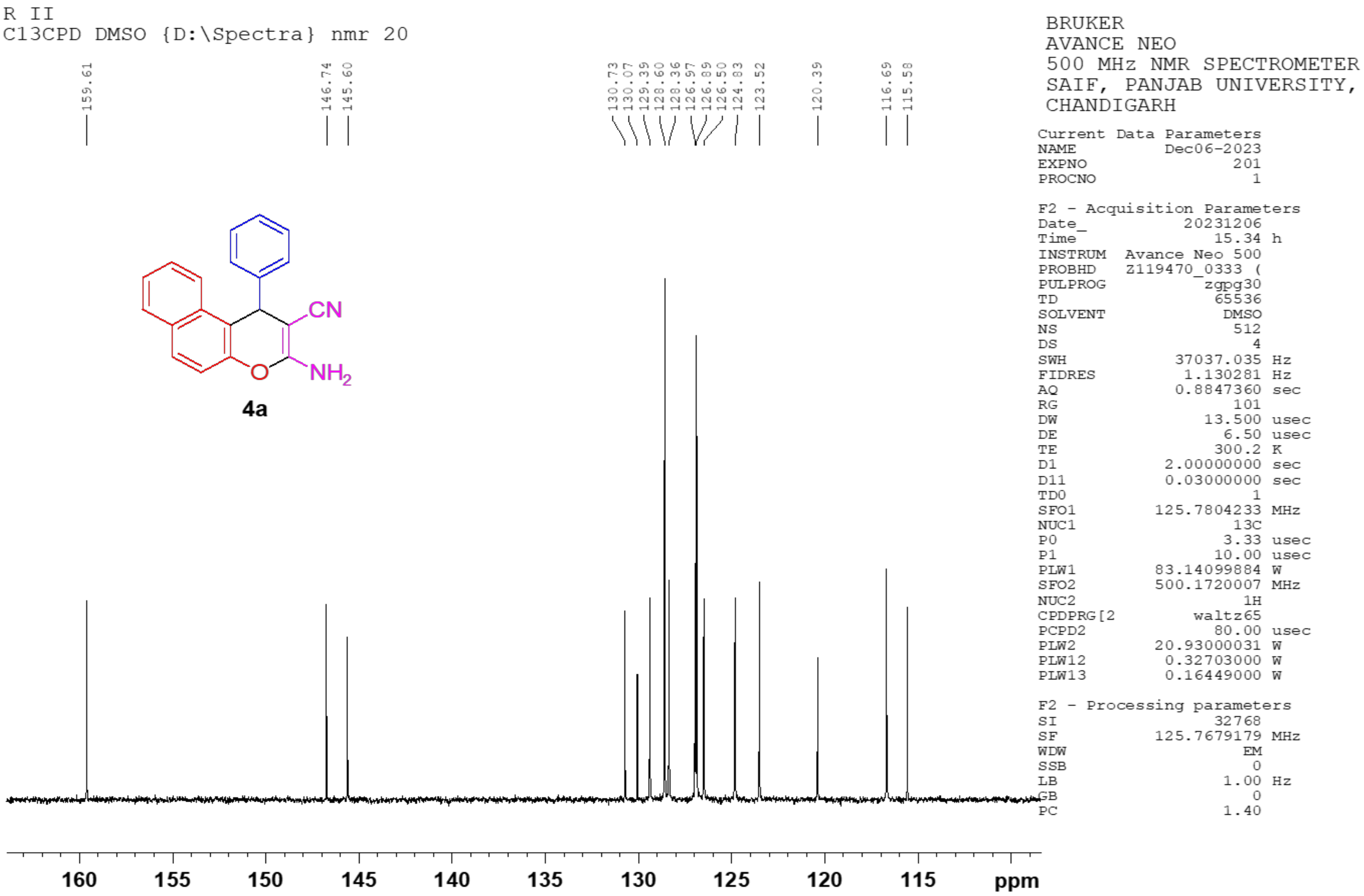

Figure S5. ^{13}C-NMR expanded spectrum of *2-Amino-4-phenyl-4H-benzo[h]chromene-3-carbonitrile* ***(4a)***.

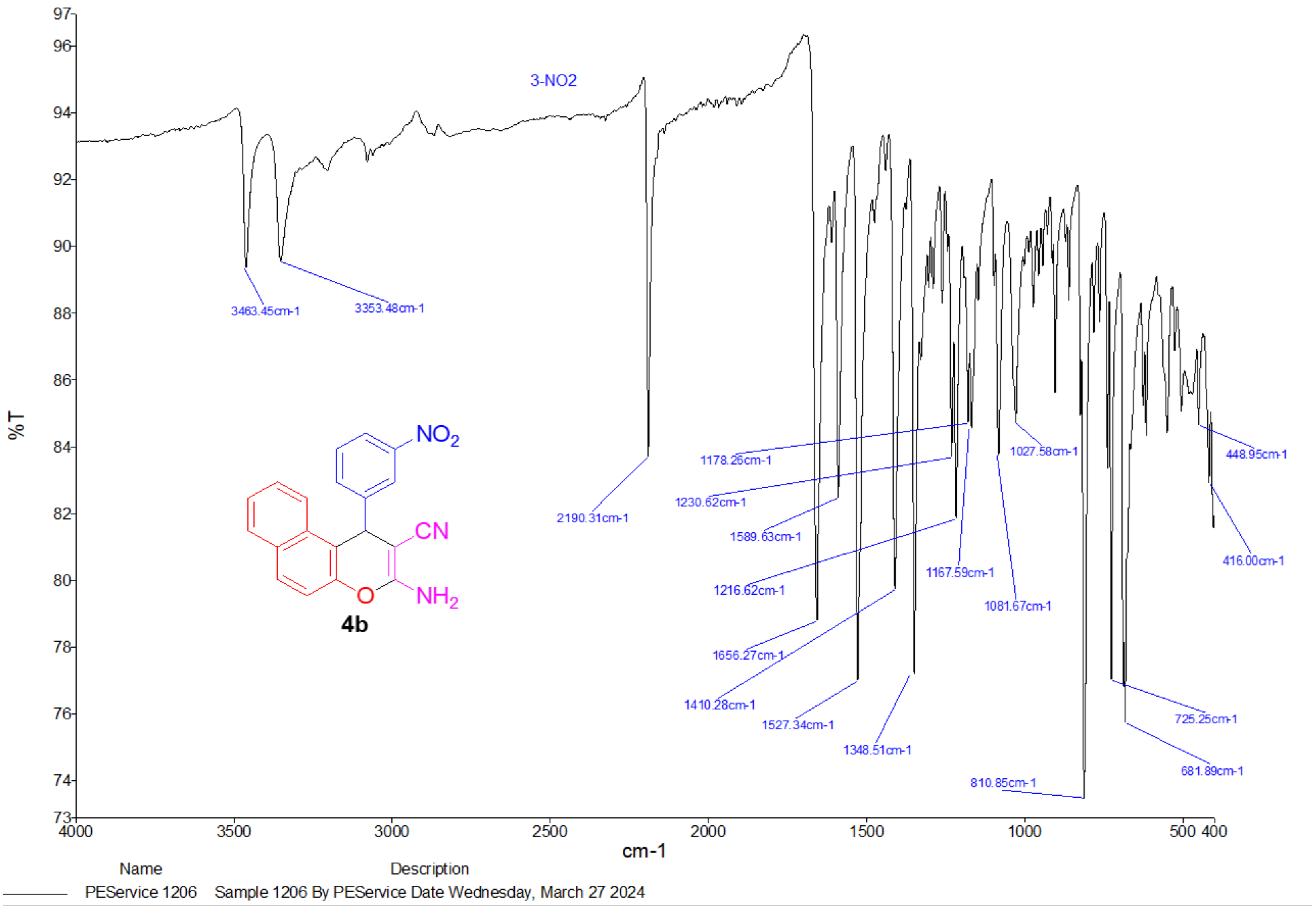

Figure S6. FT-IR spectrum of *2-Amino-4-(3-nitrophenyl)-4H-benzo[h]chromene-3-carbonitrile* ***(4b)***.

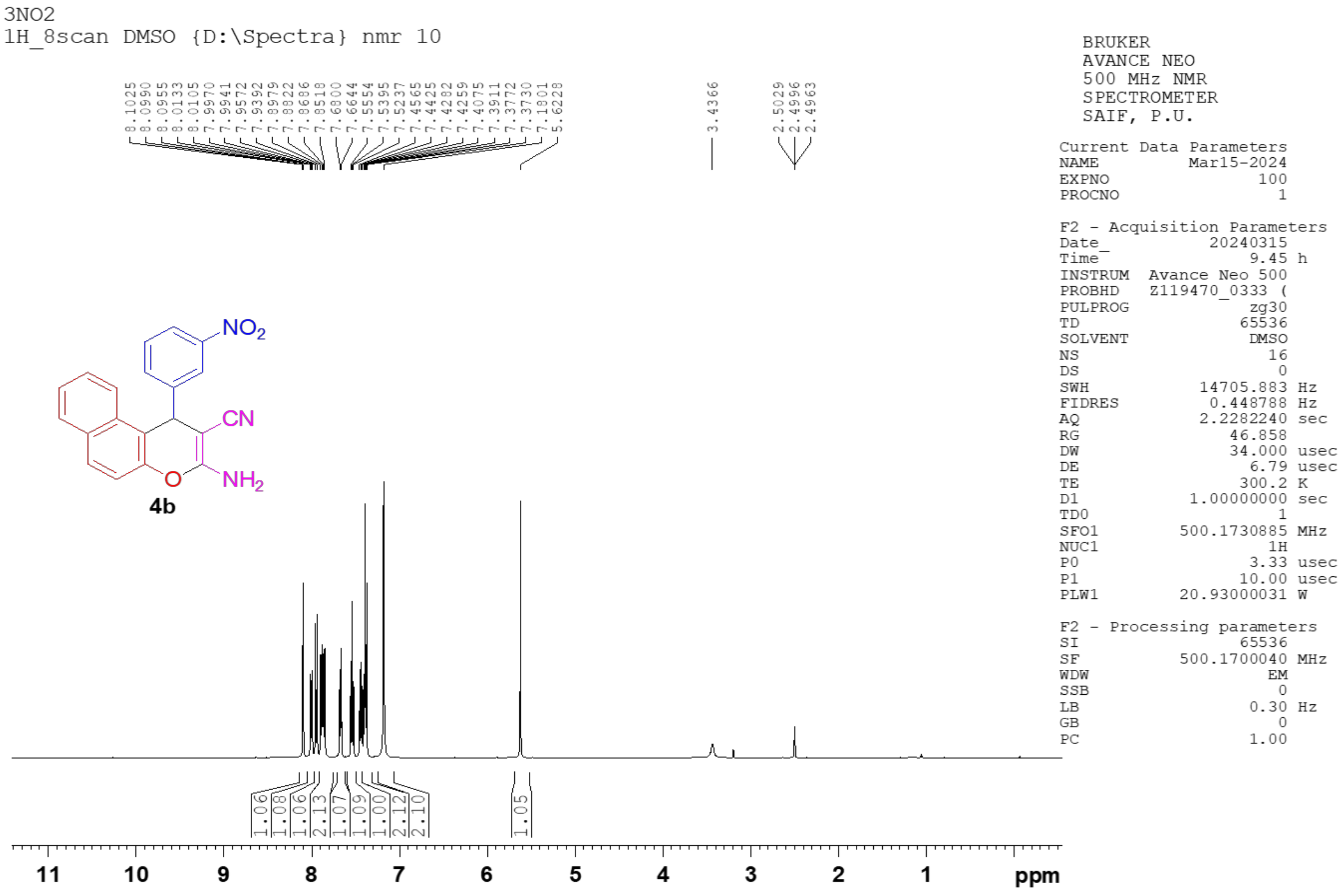

Figure S7. ^{1}H-NMR spectrum of *2-Amino-4-(3-nitrophenyl)-4H-benzo[h]chromene-3-carbonitrile* ***(4b).***

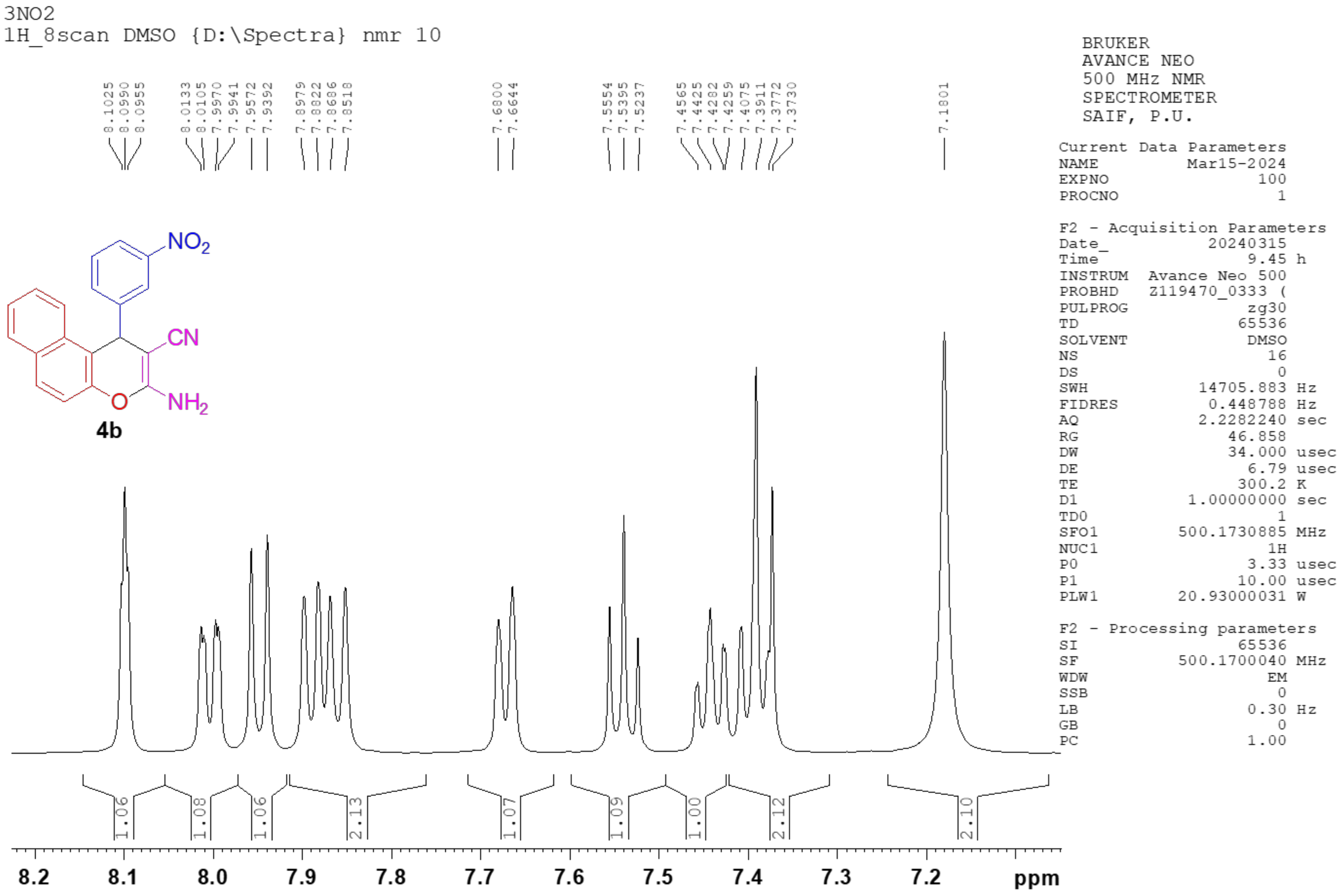

Figure S8. ^{1}H-NMR expanded spectrum of *2-Amino-4-(3-nitrophenyl)-4H-benzo[h]chromene-3-carbonitrile* ***(4b).***

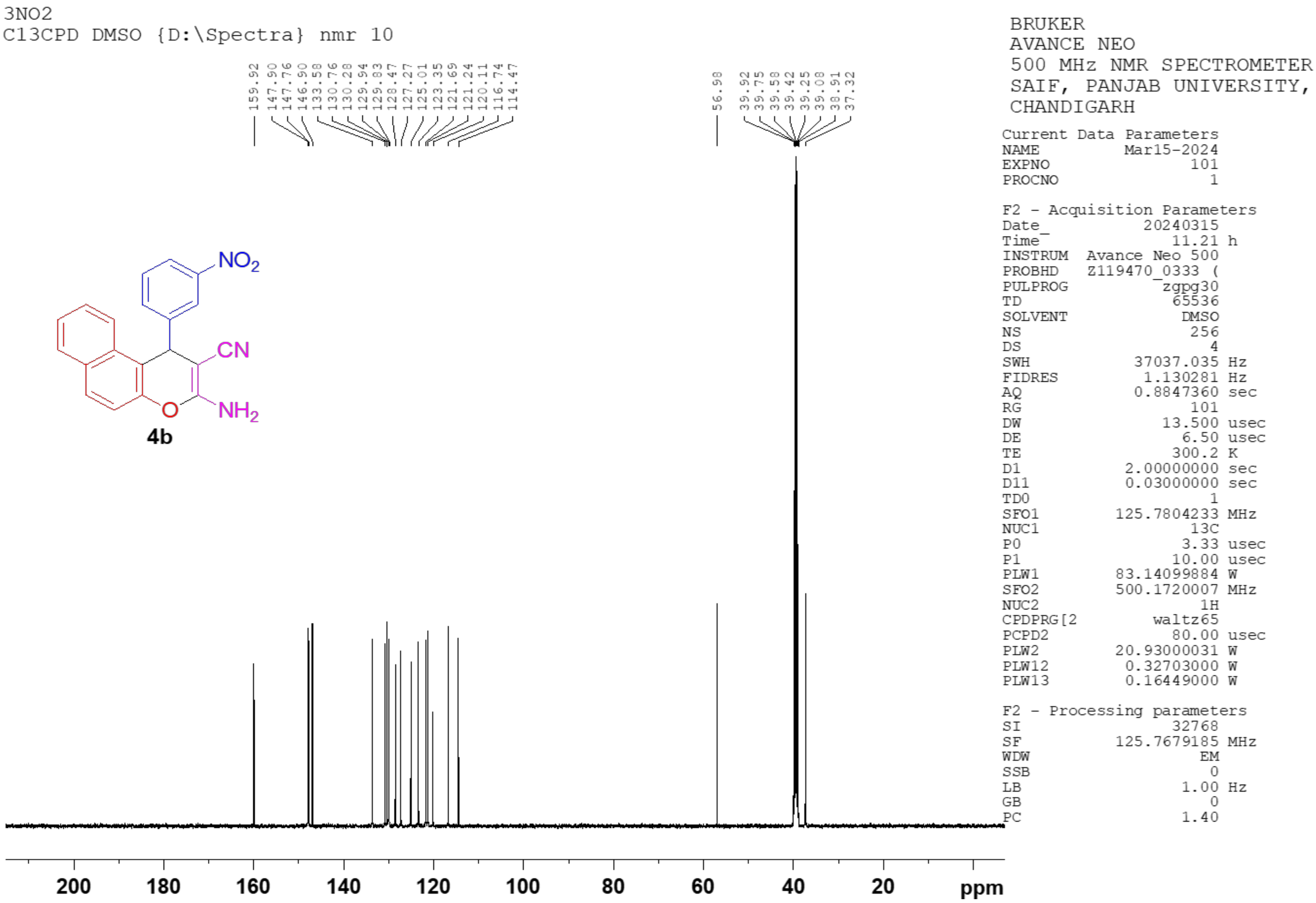

Figure S9. ^{13}C-NMR spectrum of *2-Amino-4-(3-nitrophenyl)-4H-benzo[h]chromene-3-carbonitrile* ***(4b)***.

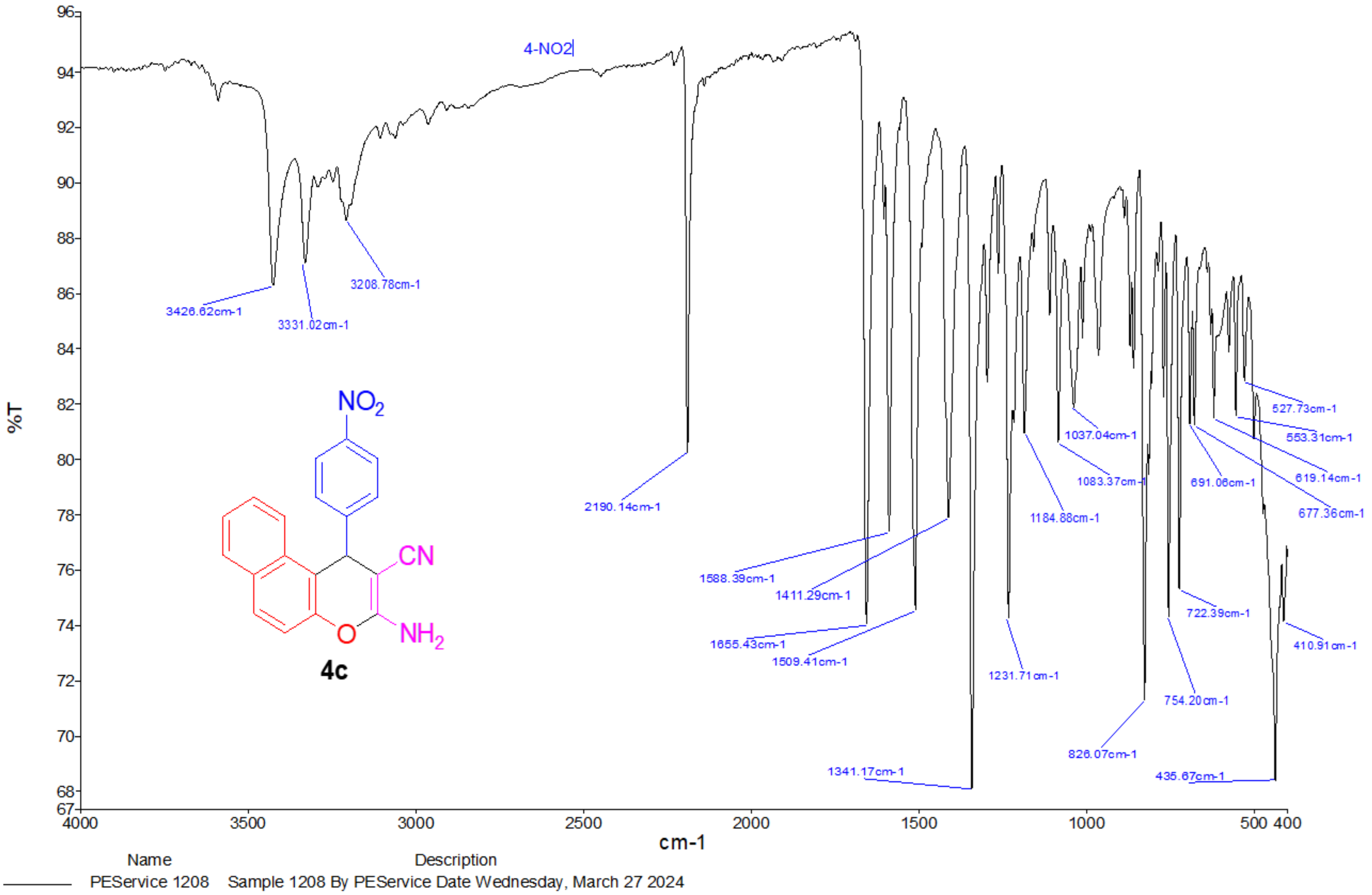

Figure S10. FT-IR spectrum of *2-Amino-4-(4-nitrophenyl)-4H-benzo[h]chromene-3-carbonitrile* ***(4c)***.

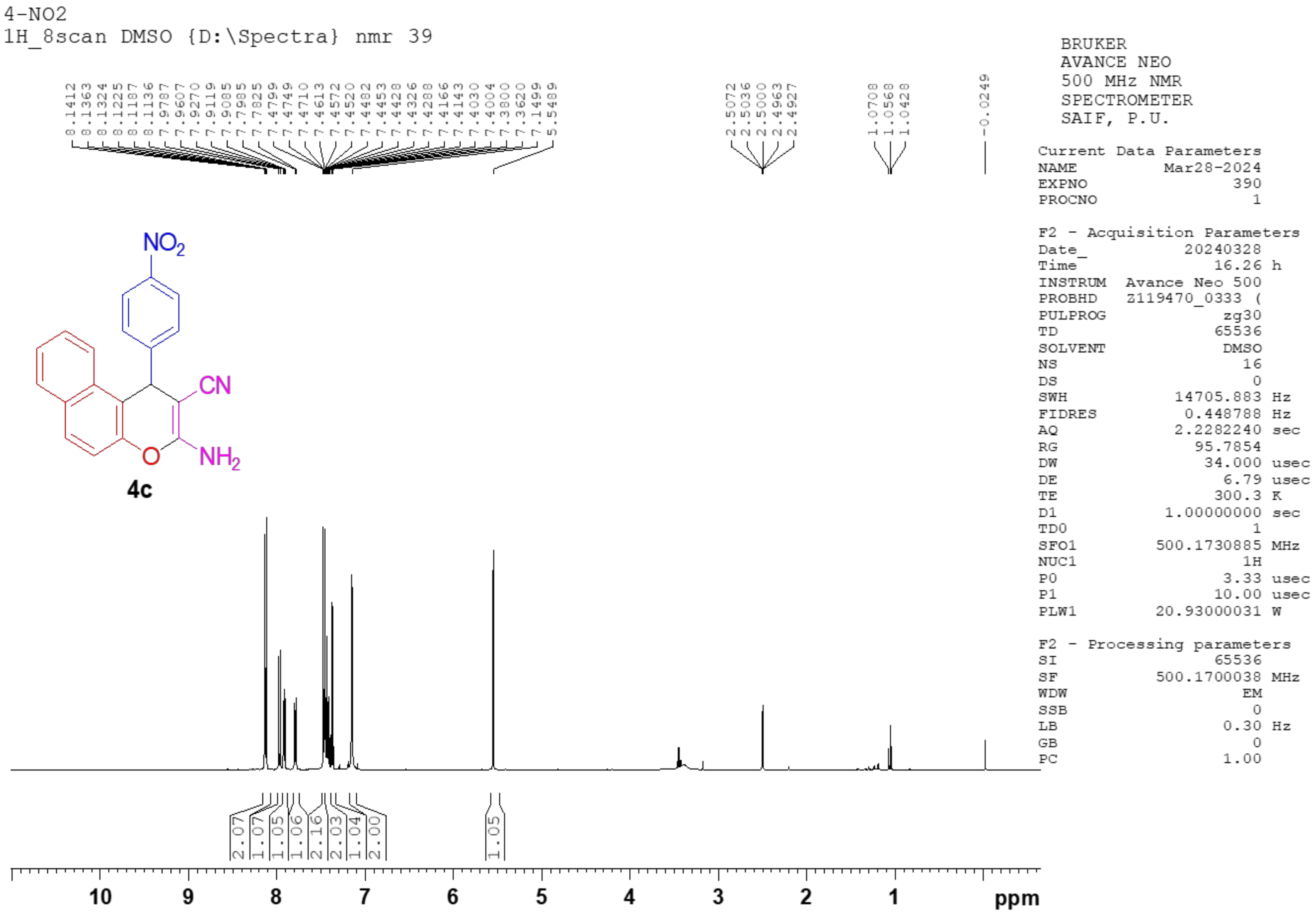

Figure S11. ^{1}H-NMR spectrum of *2-Amino-4-(4-nitrophenyl)-4H-benzo[h]chromene-3-carbonitrile* ***(4c)***.

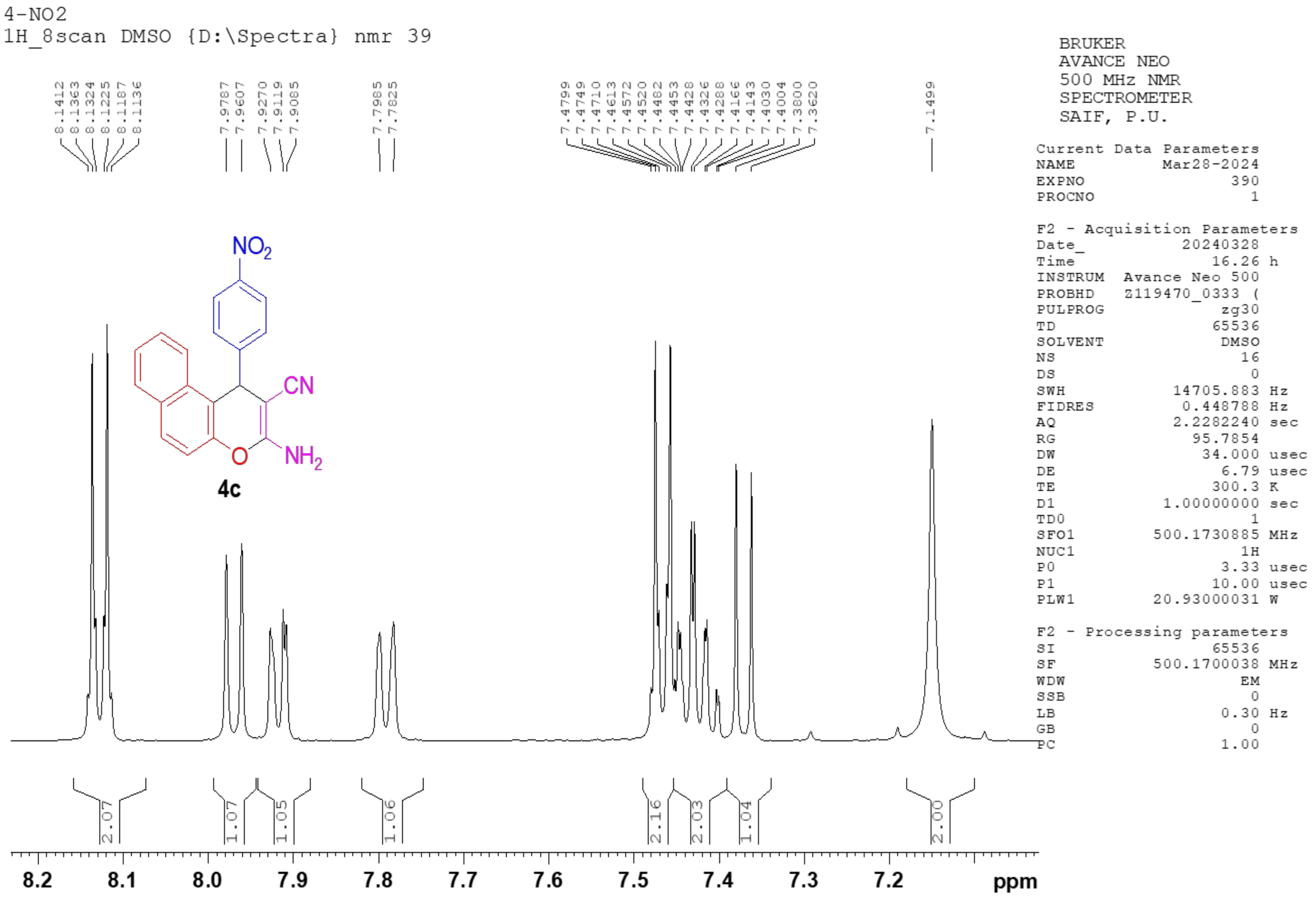

Figure S12. ^{1}H-NMR expanded spectrum of *2-Amino-4-(4-nitrophenyl)-4H-benzo[h]chromene-3-carbonitrile* ***(4c)***.

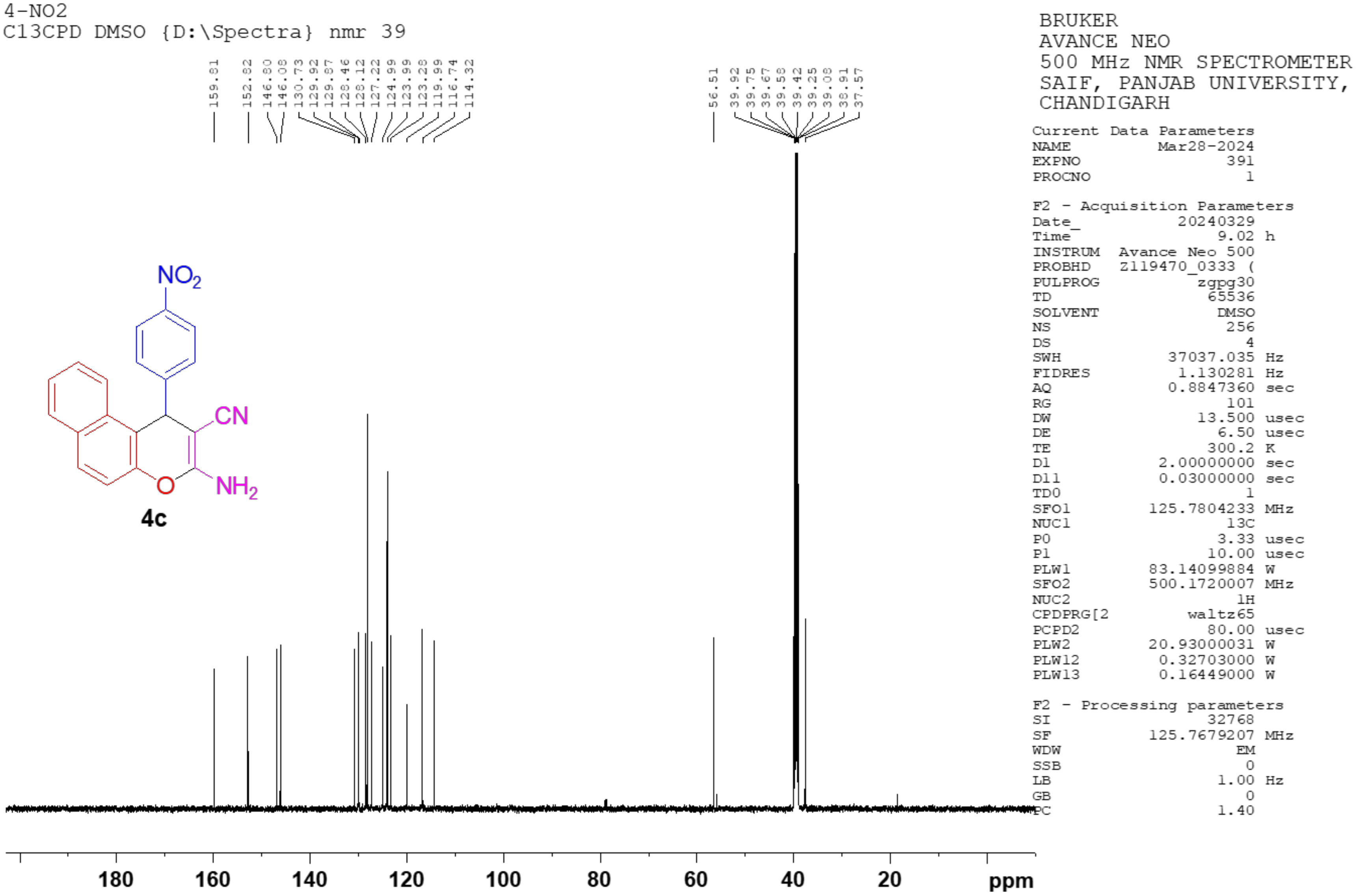

Figure S13. ^{13}C-NMR spectrum of *2-Amino-4-(4-nitrophenyl)-4H-benzo[h]chromene-3-carbonitrile* ***(4c)***.

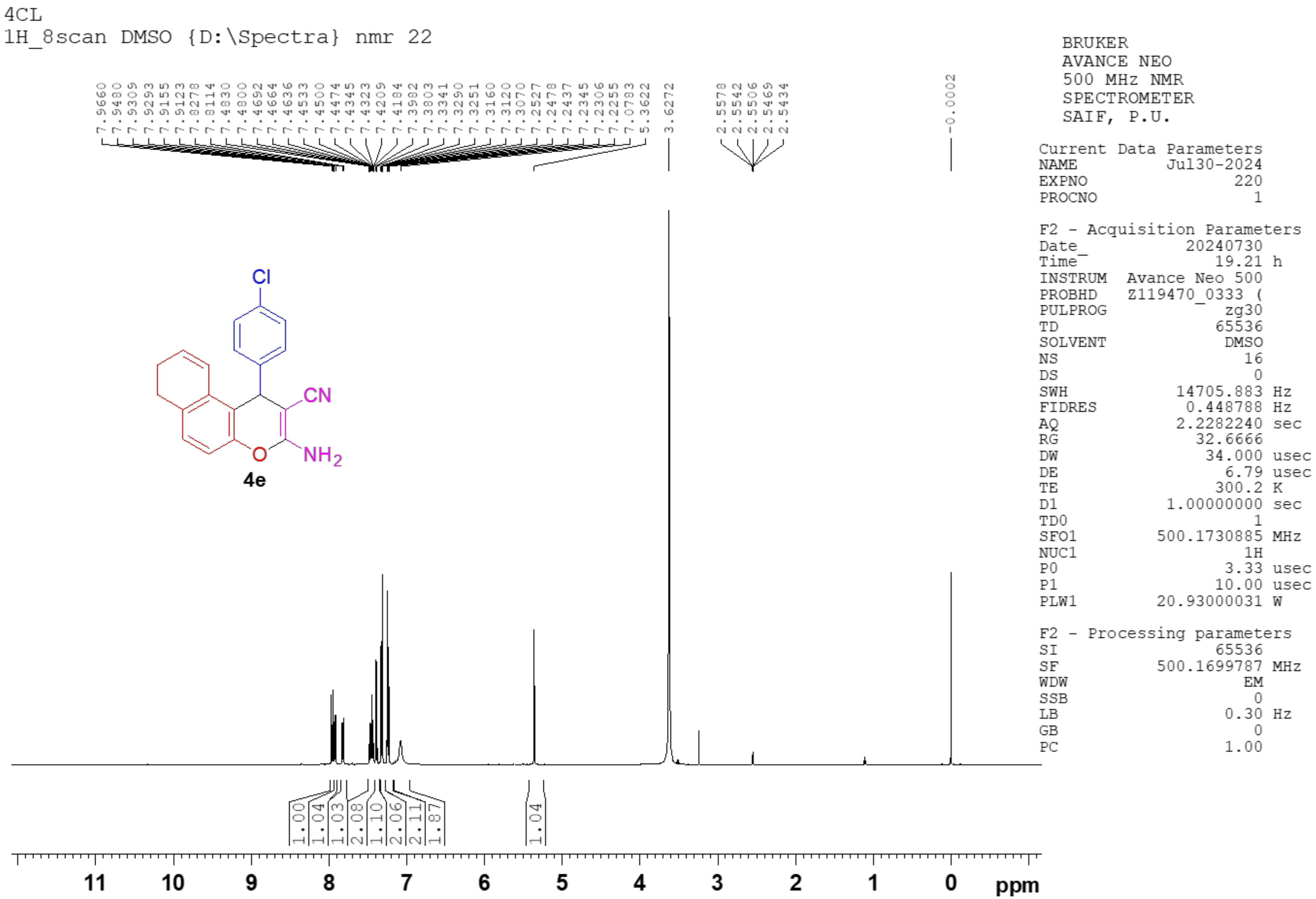

Figure S14. ^{1}H-NMR spectrum of *2-Amino-4-(4-chlorophenyl)-4-H-benzo[g] chromene-3-carbonitrile* ***(4e)***.

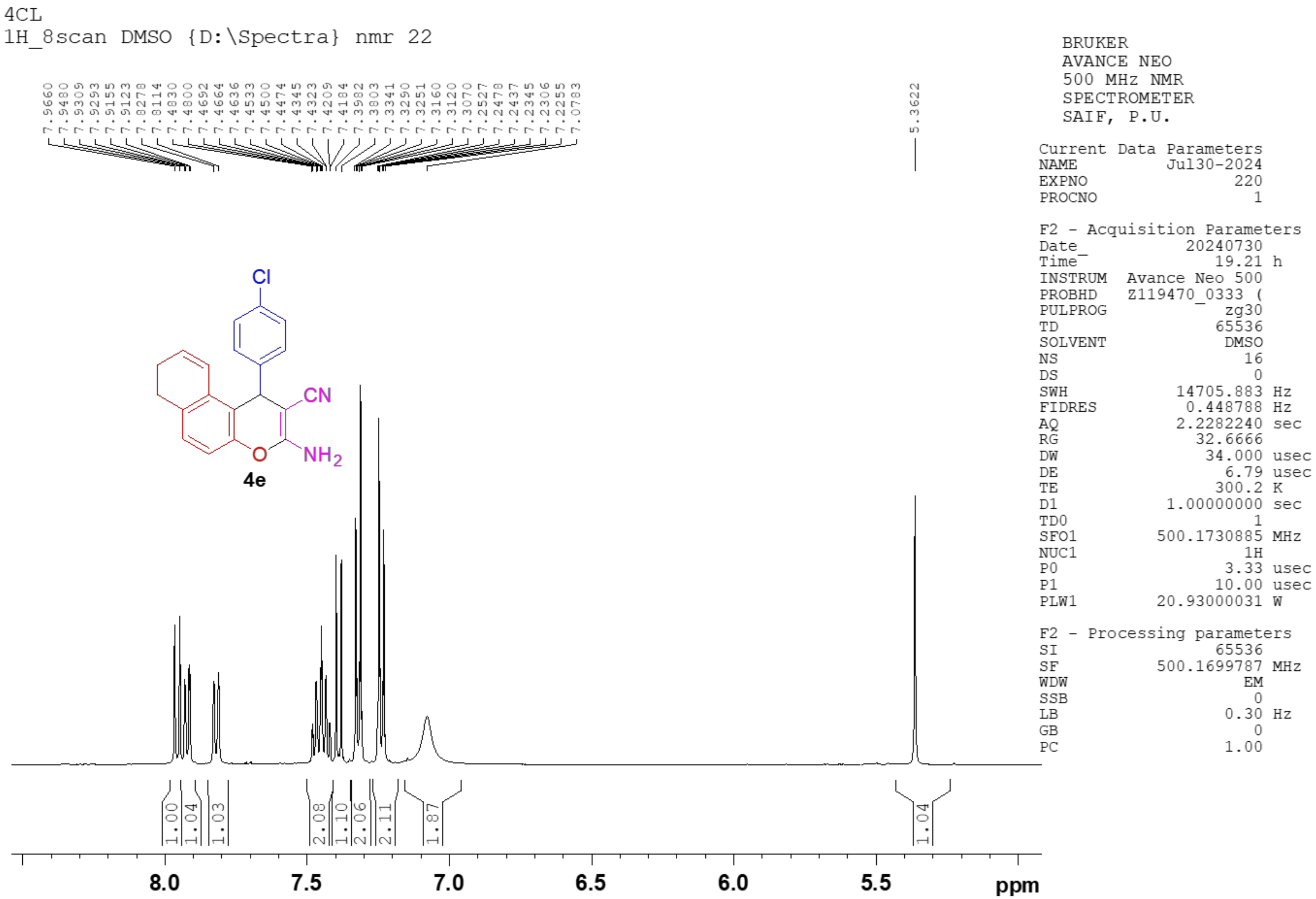

Figure S15. ^{1}H-NMR expanded spectrum of *2-Amino-4-(4-chlorophenyl)-4-H-benzo[g] chromene-3-carbonitrile* ***(4e)***.

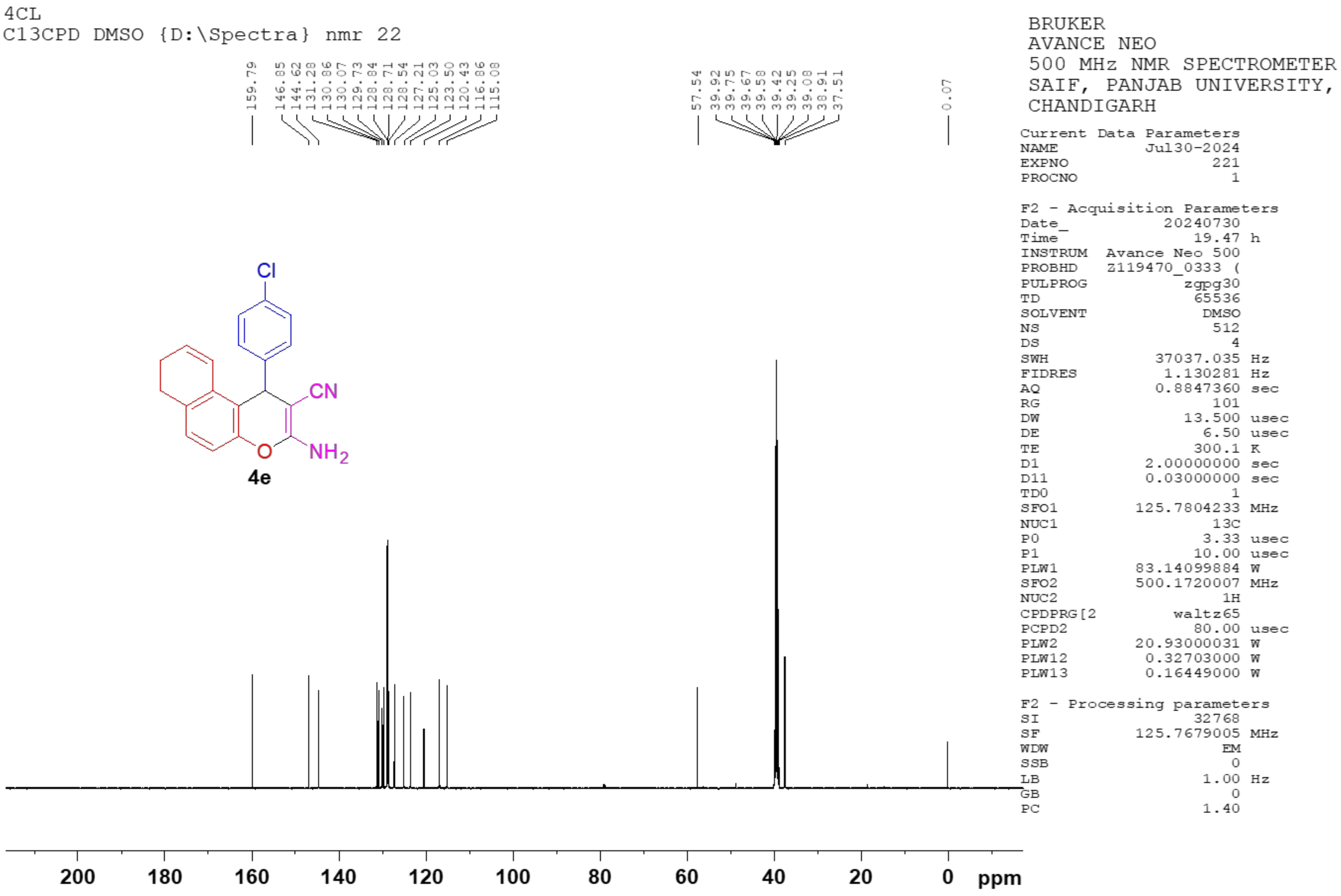

Figure S16. ^{13}C-NMR spectrum of *2-Amino-4-(4-chlorophenyl)-4-H-benzo[g] chromene-3-carbonitrile* ***(4e)***.

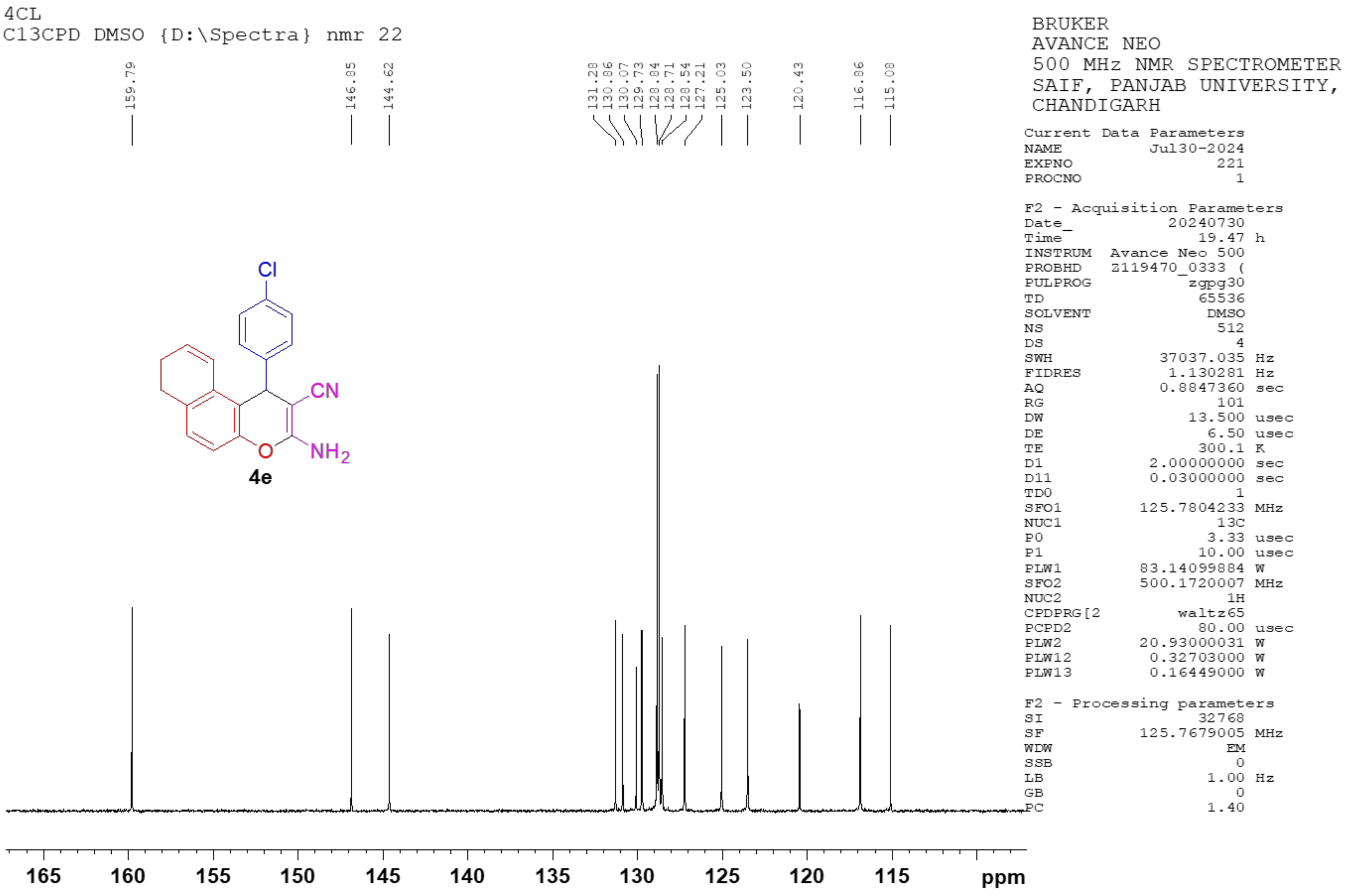

Figure S17. ^{13}C-NMR expanded spectrum of *2-Amino-4-(4-chlorophenyl)-4-H-benzo[g] chromene-3-carbonitrile* ***(4e)***.

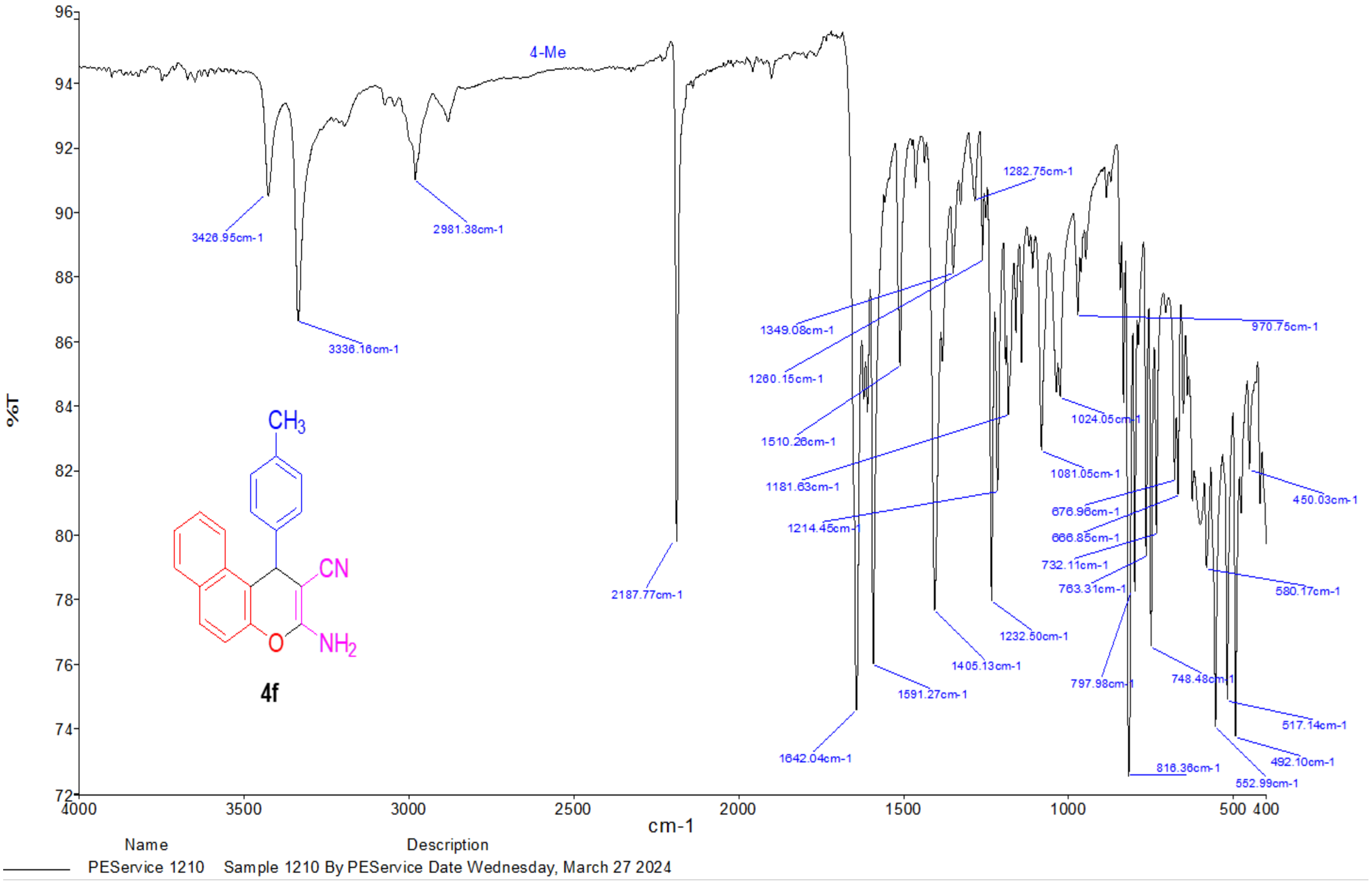

Figure S18. FT-IR spectrum of *2-Amino-4-p-tolyl-4H-benzo[h]chromene-3-carbonitrile* ***(4f)***.

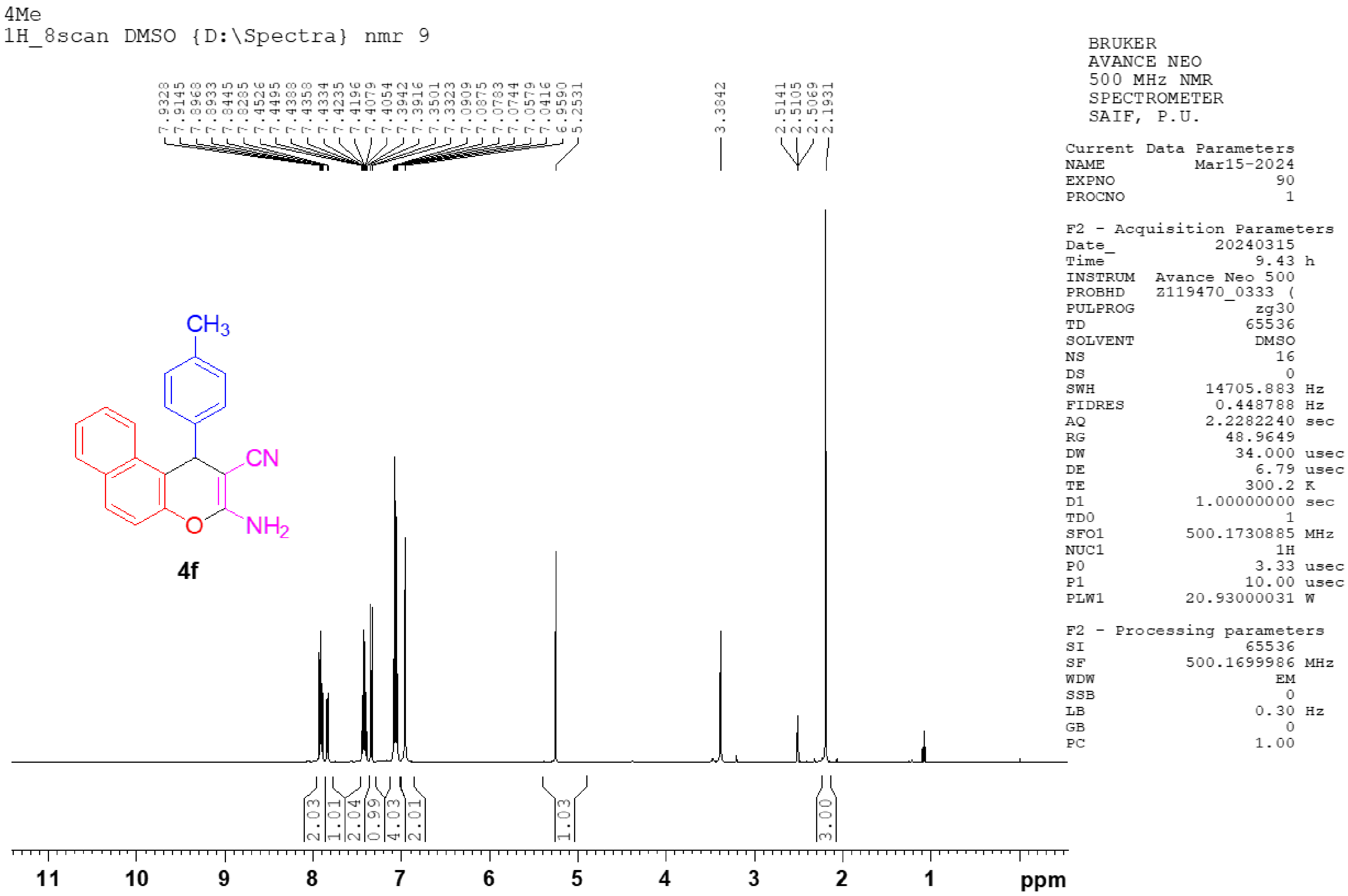

Figure S19. ^{1}H-NMR spectrum of *2-Amino-4-p-tolyl-4H-benzo[h]chromene-3-carbonitrile* ***(4f).***

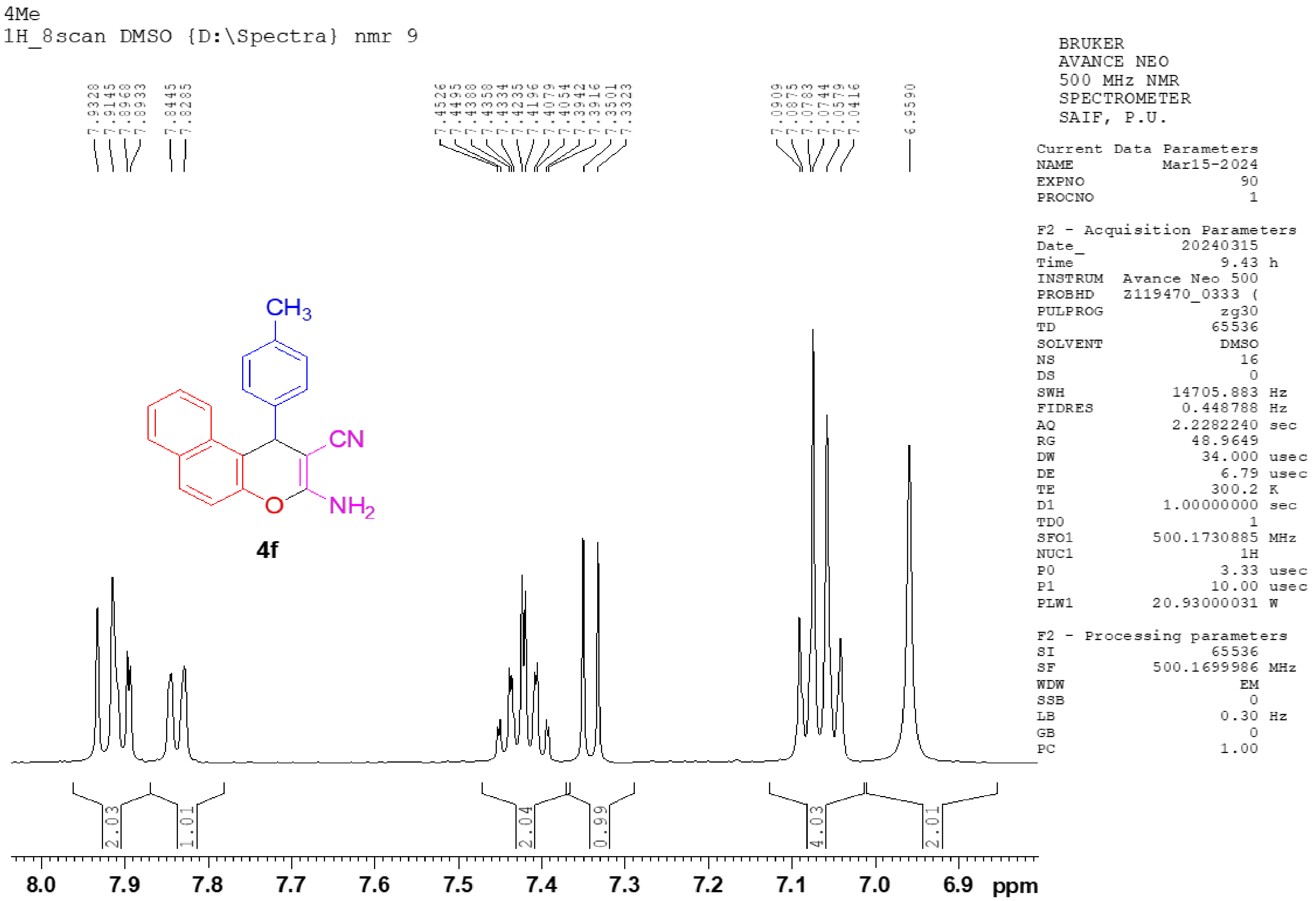

Figure S20. ^{1}H-NMR expanded spectrum of *2-Amino-4-p-tolyl-4H-benzo[h]chromene-3-carbonitrile* ***(4f)*****.**

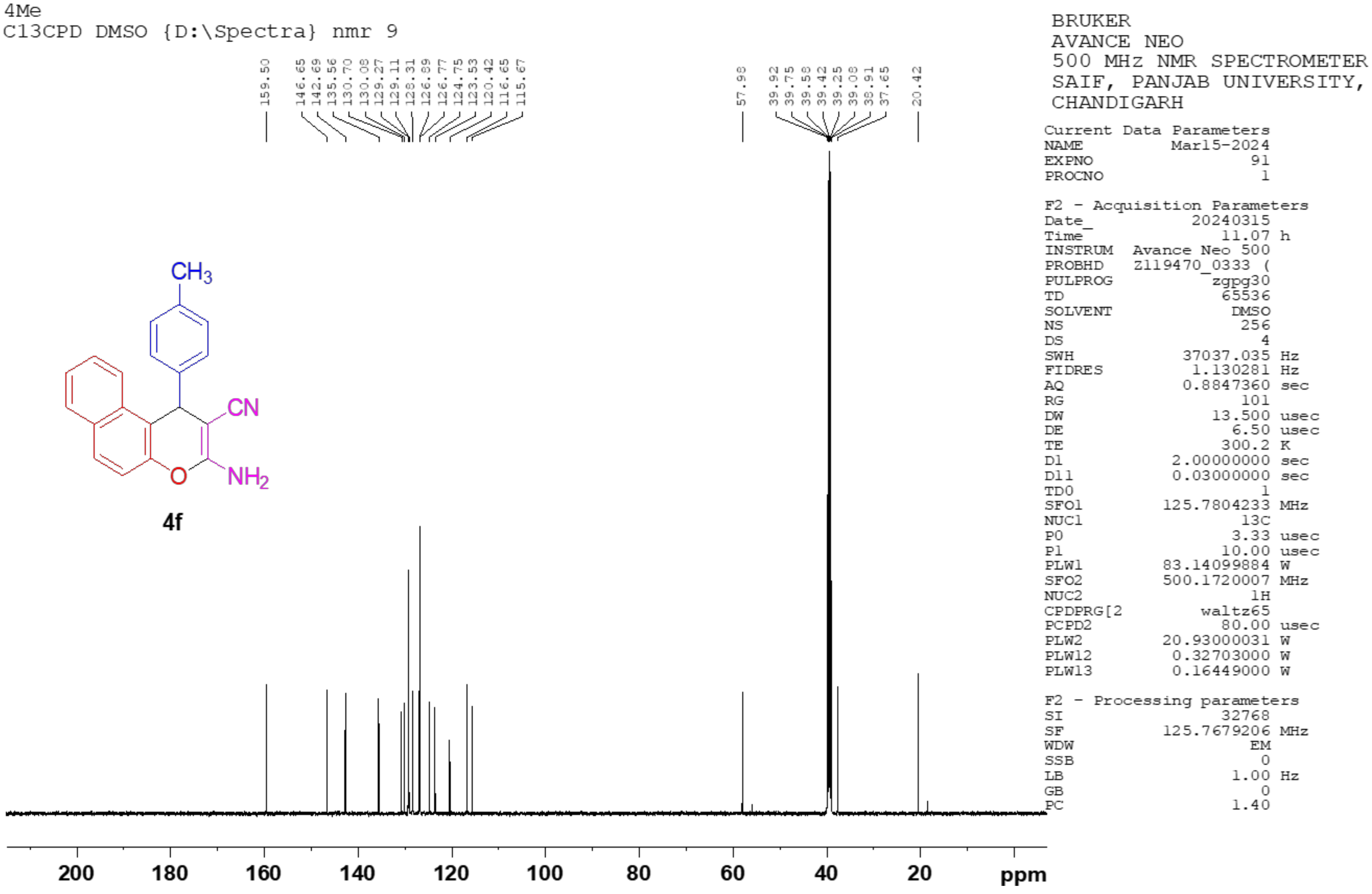

Figure S21. ^{13}C-NMR spectrum of *2-Amino-4-p-tolyl-4H-benzo[h]chromene-3-carbonitrile* ***(4f)*.**

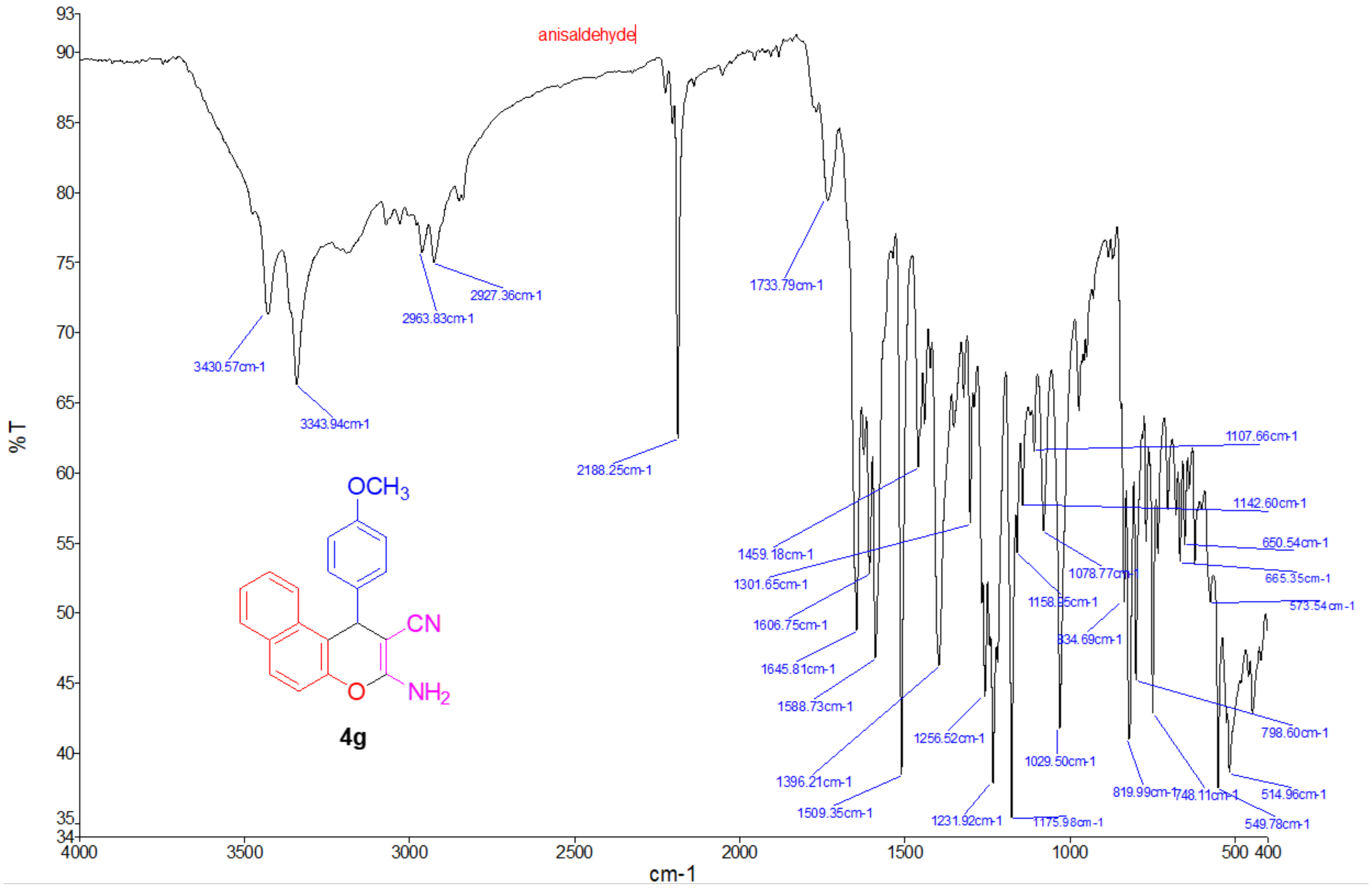

Figure S22. FT-IR spectrum of *2-Amino-4-(4-methoxyphenyl)-4-H-benzo[h] chromene-3-carbonitrile* ***(4g)*****.**

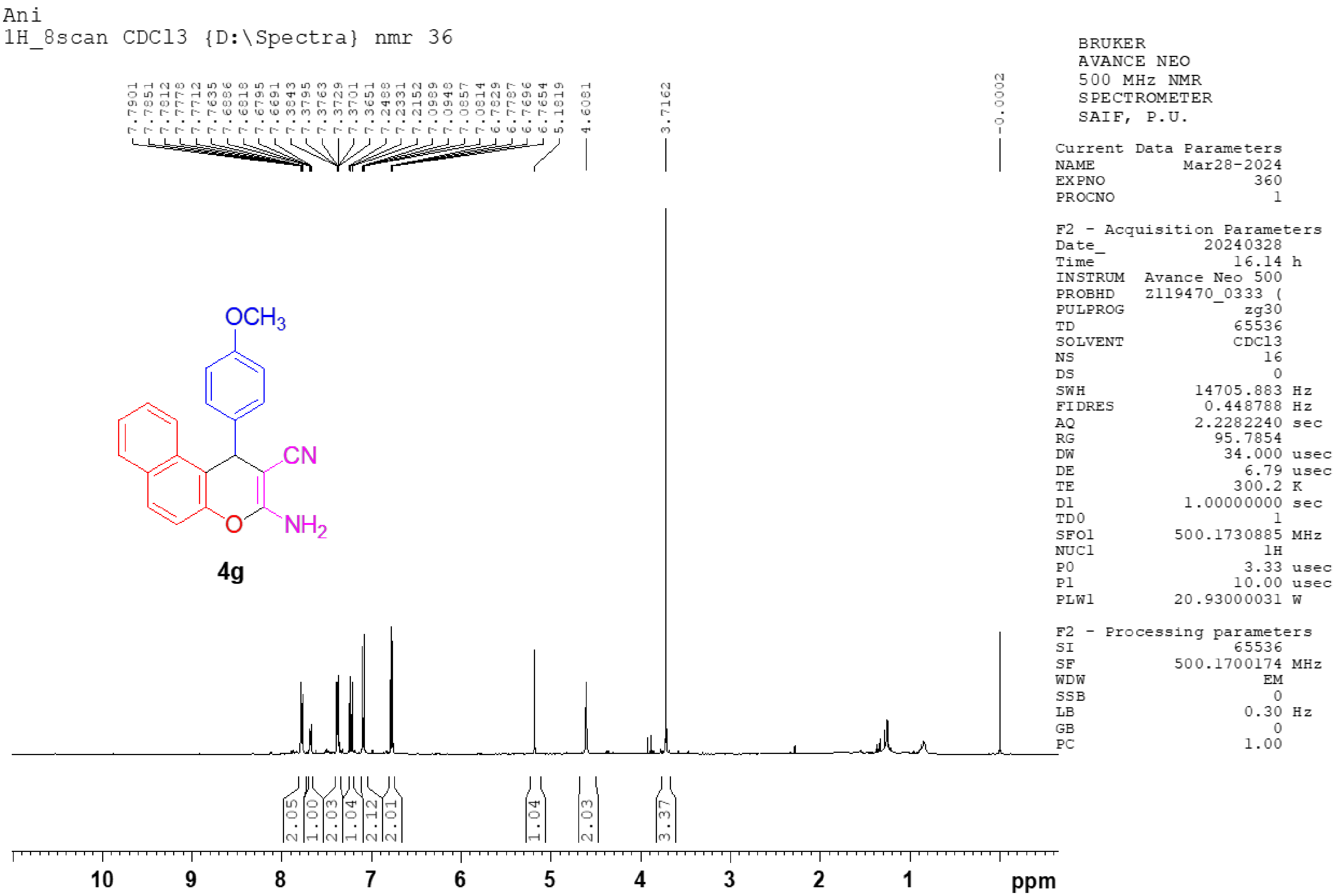

Figure S23. ^{1}H-NMR spectrum of *2-Amino-4-(4-methoxyphenyl)-4-H-benzo[h] chromene-3-carbonitrile* ***(4g).***

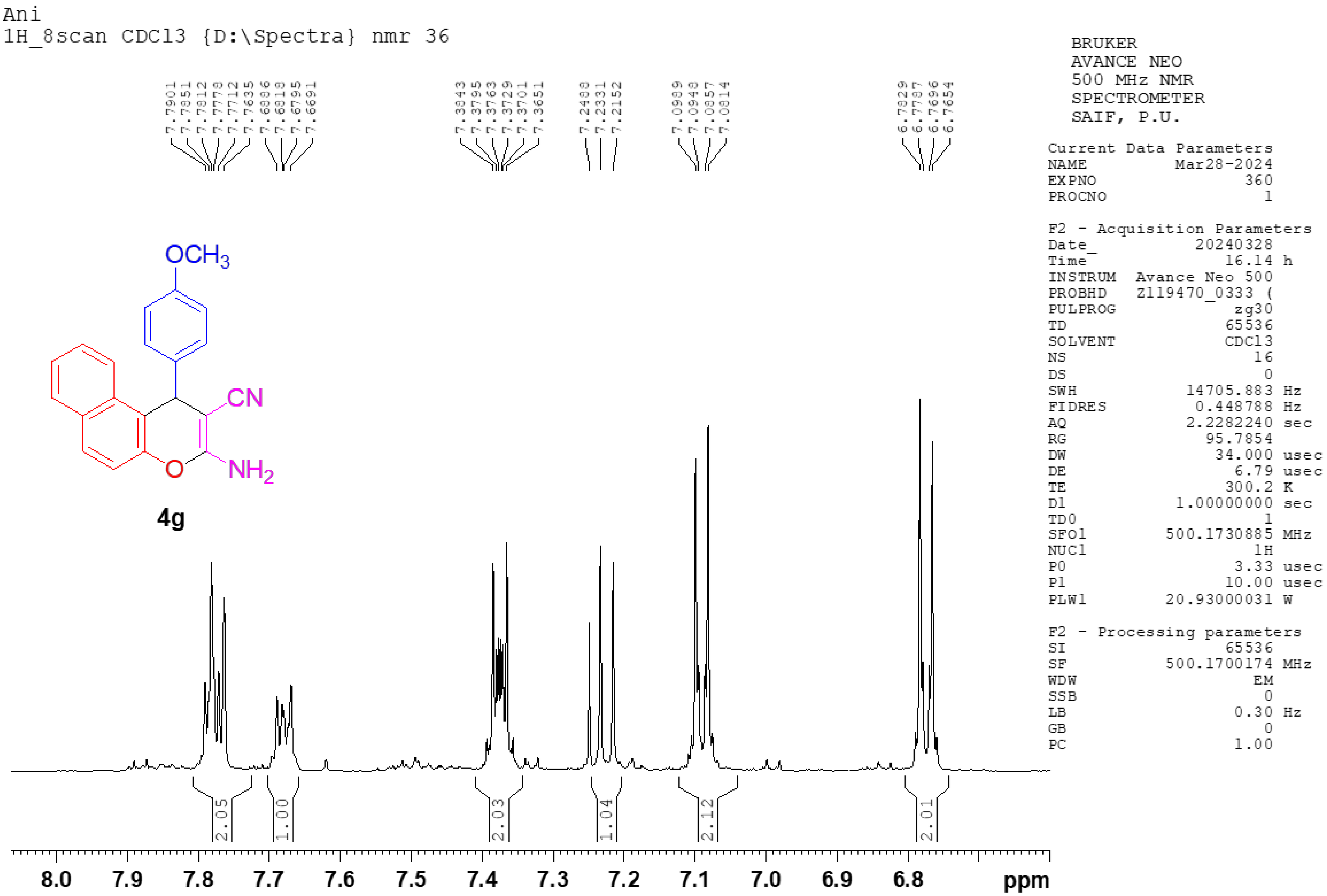

Figure S24. ^{1}H-NMR expanded spectrum of *2-Amino-4-(4-methoxyphenyl)-4-H-benzo[h] chromene-3-carbonitrile* ***(4g).***

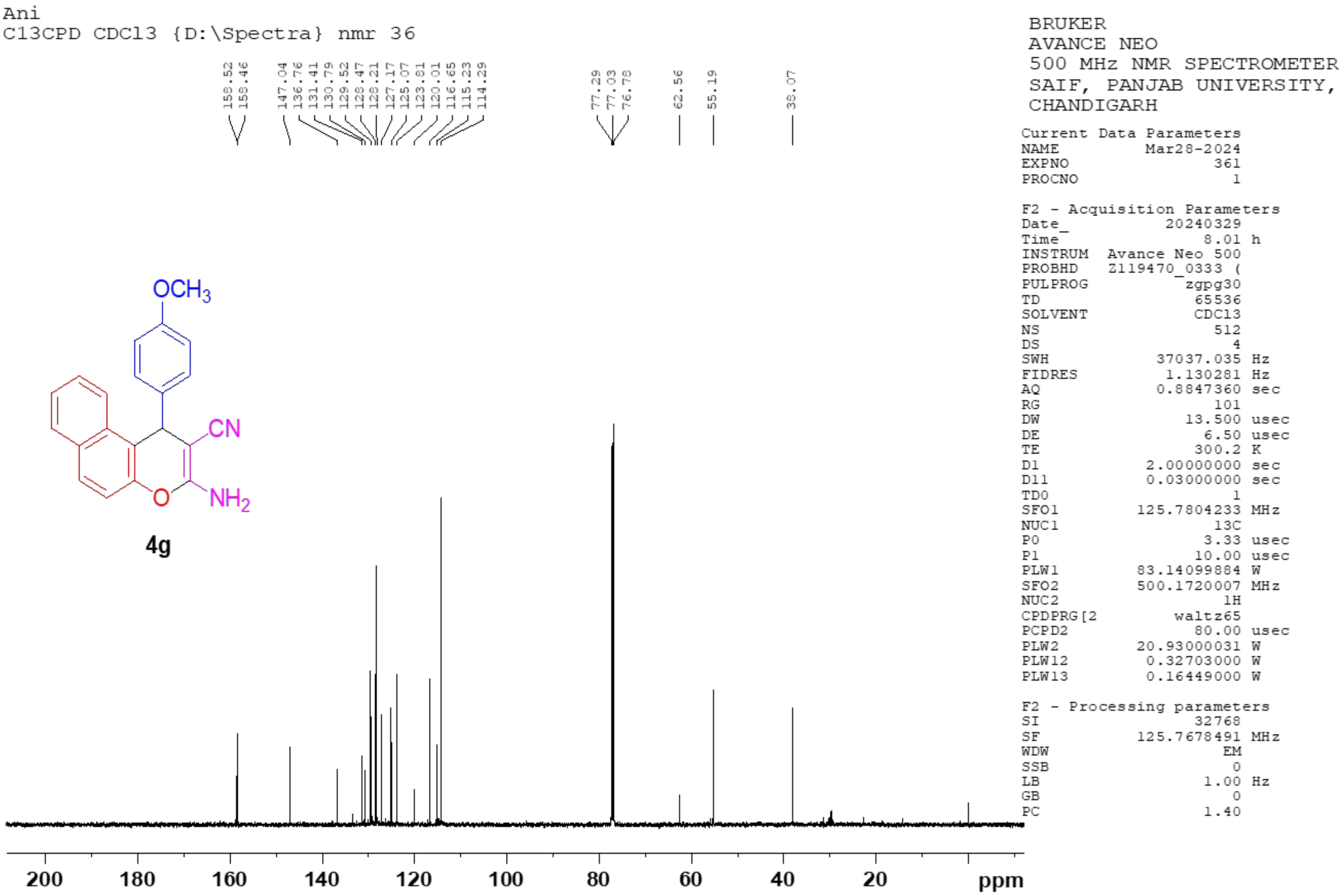

Figure S25. ^{13}C-NMR spectrum of *2-Amino-4-(4-methoxyphenyl)-4-H-benzo[h] chromene-3-carbonitrile* ***(4g)*****.**

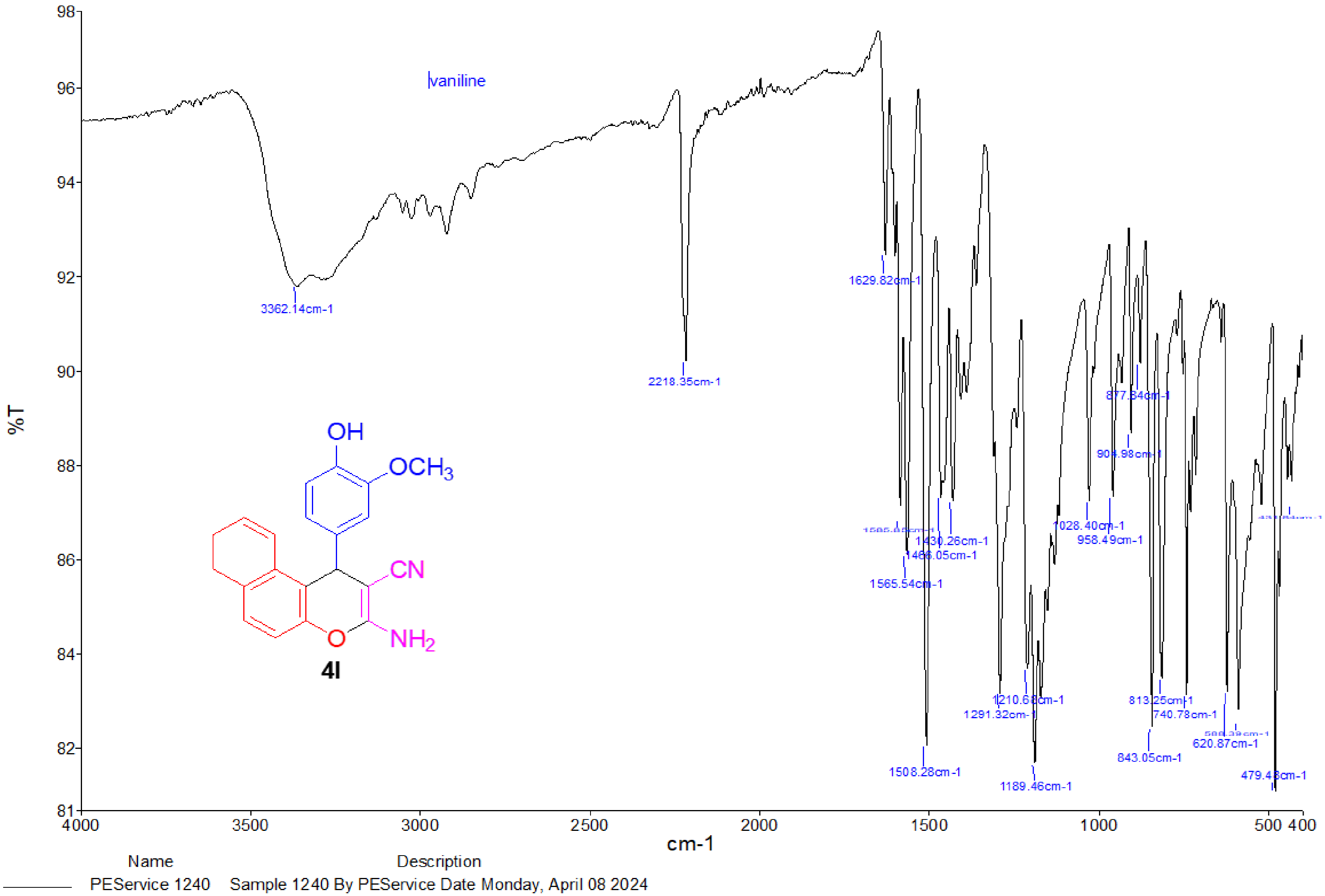

Figure S26. FT-IR spectrum of *2-Amino-1-(4-hydroxy-3-methoxyphenyl)-1H-benzo[f]chromene-2-carbonitrile* ***(4l)***.

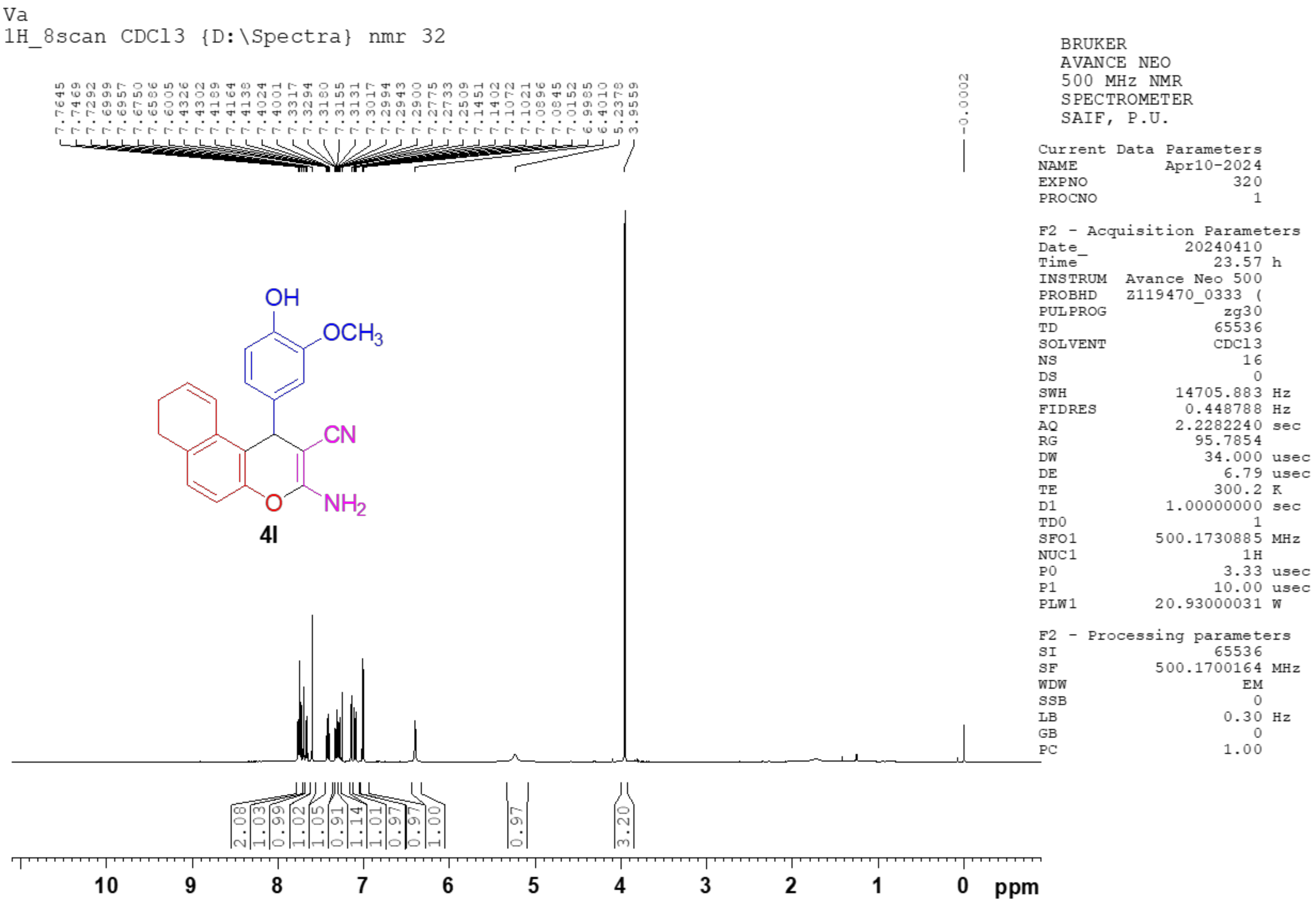

Figure S27. ^{1}H-NMR spectrum of *2-Amino-1-(4-hydroxy-3-methoxyphenyl)-1H-benzo[f]chromene-2-carbonitrile* ***(4l).***

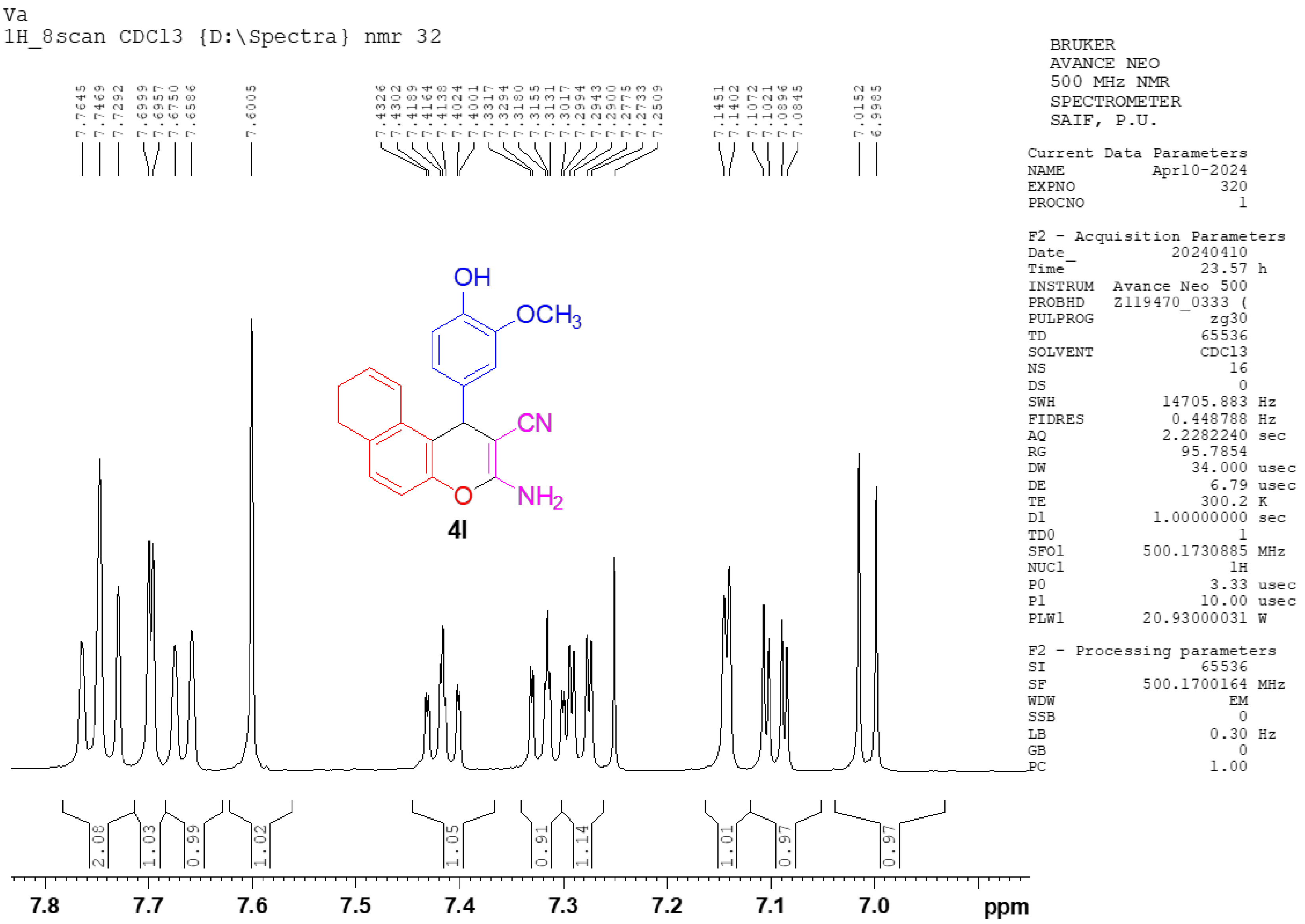

Figure S28. ^{1}H-NMR expanded spectrum of *2-Amino-1-(4-hydroxy-3-methoxyphenyl)-1H-benzo[f]chromene-2-carbonitrile* ***(4l)***.

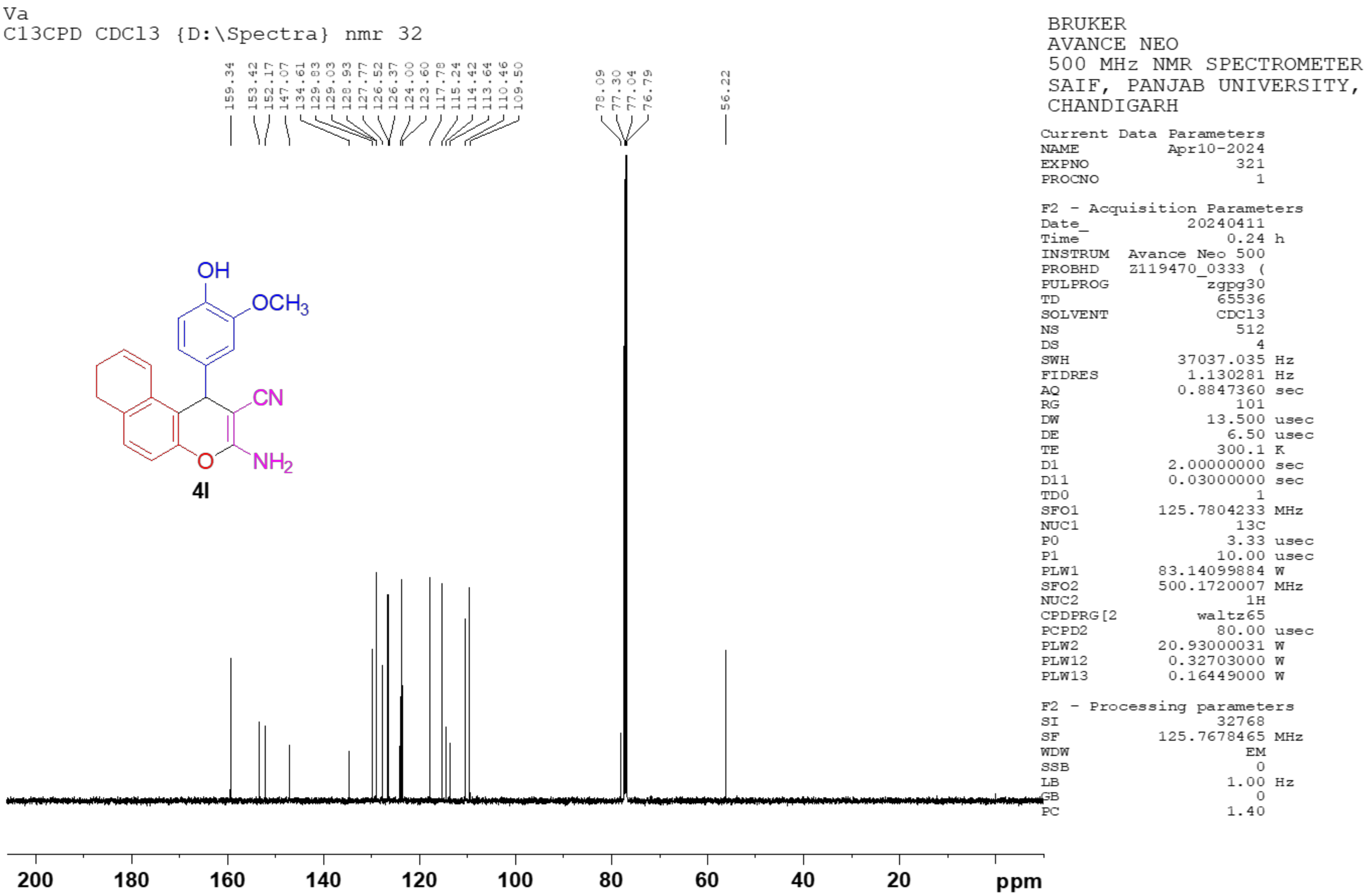

Figure S29. ^{13}C-NMR spectrum of *2-Amino-1-(4-hydroxy-3-methoxyphenyl)-1H-benzo[f]chromene-2-carbonitrile **(4l)***.
